# Acute cognitive effects of interruptions to prolonged sitting with brief standing or physical activity breaks: a systematic review and three-level meta-analysis

**DOI:** 10.1186/s12966-026-01953-6

**Published:** 2026-07-13

**Authors:** Mingnan Zhuang, Mingyue Yin, Zongnan Liu, Ningkun Xiao, Qing Yi, Zhiwei Fan, Henghao Yan, Xiupeng Li, Bitai Wu, Shuhan Qiu, Anqi Chen, Chao Xie, Chenghao Liu, Xintong Zou, Donglin Shi

**Affiliations:** 1 Sports Training College, Tianjin University of Sport, Tianjin, China; 2 School of Sport and Human Body Science, Hebei Sport University, Shijiazhuang, China; 3 Exercise & Nutrition Research Program, The Mary MacKillop Institute for Health Research, Australian Catholic University, Melbourne, VIC Australia; 4 The Faculty of Education, The National University of Malaysia, Lingkungan Ilmu, Bangi, Selangor Malaysia; 5 Department of Immunochemistry, Institution of Chemical Engineering, Ural Federal University, Yekaterinburg, Russia; 6 Laboratory for Brain and Neurocognitive Development, Department of Psychology, Institution of Humanities, Ural Federal University, Yekaterinburg, Russia; 7 Division of Sports Science and Physical Education, Tsinghua University, Beijing, China; 8 Faculty of Health Sciences and Sports, Macao Polytechnic University, Macao, S.A.R. China; 9 Institute of Sport Science, Southwest University, Chongqing, China; 10 Department of Physical Education, China University of Mining and Technology (Beijing), Beijing, China; 11 School of Physical Education and Sports Science, Hengyang Normal University, Hengyang, Hunan China; 12 East China Normal University, Shanghai, China; 13 Shandong Sport University, Jinan, China; 14 Department of Anesthesiology, The First Affiliated Hospital of Wenzhou Medical University, Wenzhou, China

**Keywords:** Prolonged sitting, Sitting interruptions, Physical activity breaks, Cognitive function, Executive function, Memory function, Three-level meta-analysis

## Abstract

**Background:**

Prolonged sitting is strongly associated with impaired cognitive function. Although traditional exercise interventions have been shown to improve cognitive outcomes, their implementation is often limited by practical constraints. In contrast, introducing interruptions to prolonged sitting with brief bouts of standing or physical activity has emerged as a more feasible alternative. However, previous research has been dominated by qualitative reviews. Moreover, some quantitative studies have not adequately addressed effect-size dependency arising from multiple outcomes and time points within the same study, and quantitative evidence regarding specific cognitive domains and interruption-protocol parameters remains limited.

**Methods:**

Following the PRISMA guidelines, this study systematically searched PubMed, Web of Science, the Cochrane Library, Embase, and Scopus for randomized crossover trials published up to January 2026 that examined the effects of brief standing or physical activity breaks during prolonged sitting on cognitive function. A three-level random-effects meta-analysis was conducted to pool effect sizes, with cluster-robust variance estimation used to account for effect-size dependency resulting from multiple outcomes and time points within the same study. In addition, subgroup and meta-regression analyses were performed to investigate potential moderating factors. Risk of bias was assessed using the Risk of Bias 2 tool, and the certainty of the evidence was evaluated according to the GRADE framework.

**Results:**

A total of 21 randomized crossover trials involving 433 participants were included. Compared with prolonged sitting, sitting interruptions involving standing or physical activity were associated with improvements in executive function (*k* = 16 studies; Hedges’ *g* = 0.18; 95% *CI*: 0.03 to 0.32; *p* = 0.016) and memory function (*k* = 10 studies; Hedges’ *g* = 0.37; 95% *CI*: 0.13 to 0.61; *p* = 0.003). However, no clear evidence of improvement was found for global cognition (*k* = 2 studies; Hedges’ *g* = 0.57; 95% *CI*: −0.20 to 1.34; *p* = 0.122), information processing (*k* = 4 studies; Hedges’ *g* = 0.32; 95% *CI*: −0.21 to 0.86; *p* = 0.221), or attention (*k* = 5 studies; Hedges’ *g* = − 0.08; 95% *CI*: −0.38 to 0.22; *p* = 0.575). The certainty of evidence was low for executive function, memory function, information processing, and attention, and very low for global cognition. Exploratory subgroup analyses suggested that cognitive benefits were more pronounced in older adults and in individuals with overweight or obesity. Interruptions involving walking showed relatively consistent beneficial effects, whereas low-intensity activity was associated with particularly marked improvements in memory function *(p for subgroup difference < 0.05*). Meta-regression indicated that a higher interruption frequency was associated with greater improvements in executive function, whereas improvements in memory function were associated with a lower interruption frequency and longer experimental exposure. Overall, however, the certainty of evidence for most outcomes was rated as low or very low.

**Conclusions:**

Introducing brief standing or physical activity breaks during prolonged sitting may be associated with small acute improvements in executive function and memory function, although the certainty of evidence for these outcomes remains low. Evidence for global cognition, information processing, and attention remains insufficient and inconsistent, with very low certainty for global cognition. Observed differences across participant characteristics and interruption parameters should be interpreted as exploratory and are not sufficient to support definitive prescriptive recommendations. From a feasibility perspective, low-intensity walking may represent a practical option, but its relative efficacy requires confirmation in larger, higher-quality studies.

**Trial registration:**

CRD420251273873; registered 26 December 2025.

**Graphical abstract:**

**Figure:**
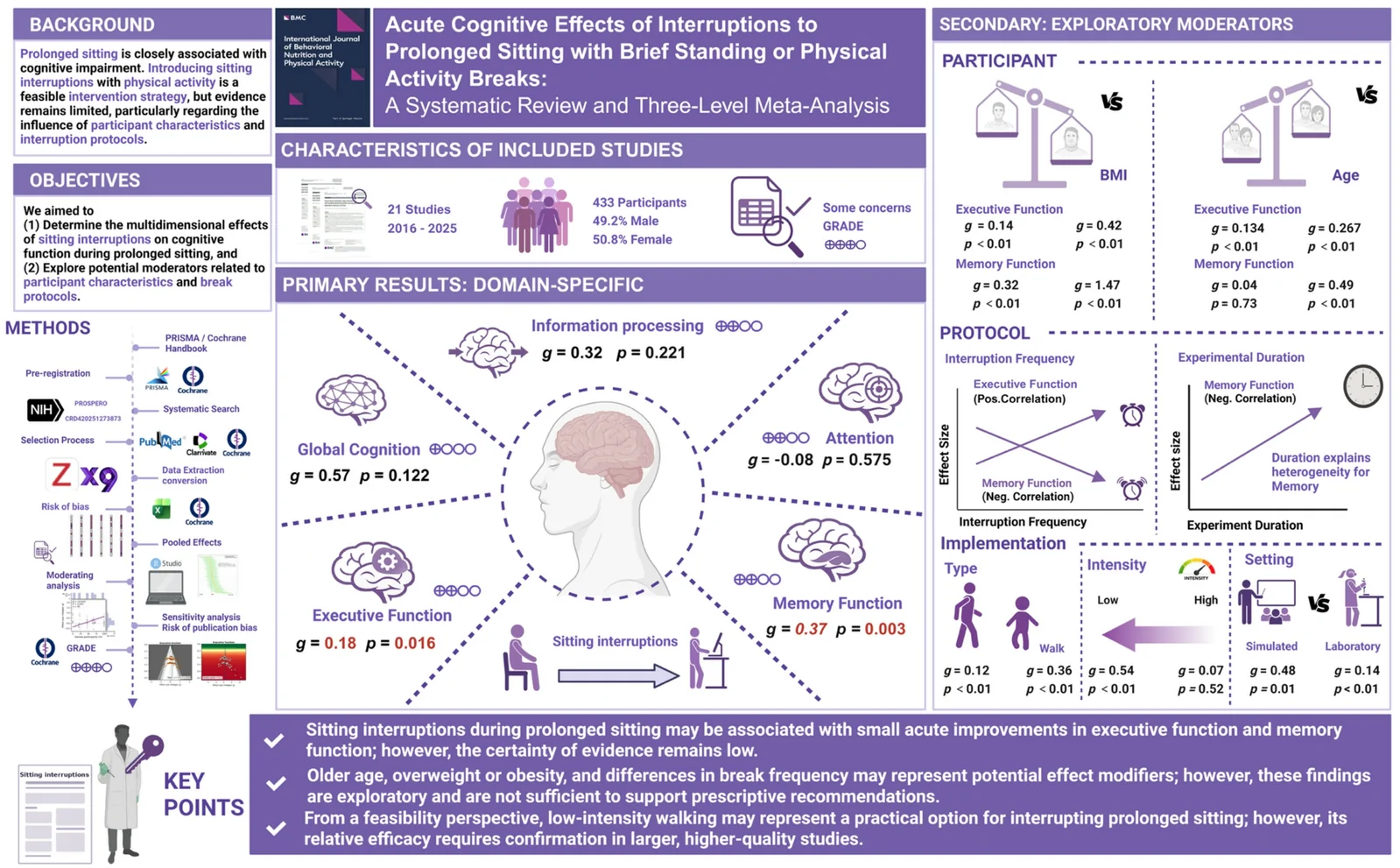

**Supplementary Information:**

The online version contains supplementary material available at 10.1186/s12966-026-01953-6.

## Background

As the global population ages at an accelerating rate, the social and healthcare burden associated with cognitive decline and related disorders continues to increase, emerging as a major public health challenge that requires urgent attention [1]. Sedentary behavior is defined as any waking behavior, such as sitting, reclining, or lying down, that involves an energy expenditure of 1.5 metabolic equivalents (METs) or less [2]. In increasingly digitalized work and study environments, sedentary behavior has become a dominant component of daily life. Epidemiological surveys indicate that adults worldwide spend at least half of their waking hours sedentary, with some studies reporting proportions as high as approximately two-thirds [3–8]. This level of exposure approaches, and in some cases exceeds, the sedentary thresholds (e.g., > 8 h/day) identified in previous studies as being associated with an increased risk of all-cause and premature mortality [9, 10]. In recent years, converging evidence from animal studies [11, 12], cohort studies [13–15], and systematic reviews [16–18] suggests that the health risks associated with sedentary behavior extend beyond cardiometabolic and cardiovascular outcomes to include cognitive function and brain health. Importantly, accumulating evidence indicates that the health consequences of sedentary behavior are determined not only by its total duration but also by how it is accumulated. Prolonged, uninterrupted sitting appears to be particularly detrimental compared with sedentary time that is regularly interrupted by brief bouts of standing or physical activity [19]. Because sedentary behavior is often unavoidable in real-world contexts such as workplaces and educational settings, identifying scalable, low-cost, and sustainable strategies that interrupt prolonged sitting, rather than eliminate sedentary time altogether, has become an important priority for preserving cognitive health in both clinical practice and public health.

Numerous studies have shown that various forms of traditional structured exercise can improve cognitive function [20–22]. However, the practical implementation of such exercise programs is often constrained by limited time and by the need for specialized equipment or designated recreational facilities, making long-term adherence and consistent participation difficult to achieve [23, 24]. In contrast, a growing body of evidence suggests that interrupting prolonged sedentary behavior with brief bouts of light-to-moderate physical activity, such as walking, stair climbing, or other lifestyle-integrated movements, may be more easily incorporated into daily routines and may help mitigate the acute adverse effects of prolonged sitting [25, 26]. Experimental studies have shown that prolonged sedentary periods can reduce cerebral arterial blood flow velocity by approximately 5%–6% [27] and are associated with significantly higher error rates on certain cognitive tasks, with some studies reporting increases of up to approximately 56% [28]. From a mechanistic perspective, introducing sitting interruptions may help maintain or improve acute cognitive performance by enhancing endothelial shear stress and cerebral perfusion [29], as well as by stimulating the release of brain-derived neurotrophic factor and eliciting anti-inflammatory responses [30]. Accordingly, incorporating brief bouts of physical activity to interrupt prolonged sitting is increasingly recognized as a feasible and potentially beneficial behavioral strategy.

This perspective is consistent with the evolving direction of contemporary public health guidelines. The WHO Guidelines on Physical Activity and Sedentary Behaviour (2020) [31] no longer emphasize the requirement that each bout of exercise must last at least 10 min. Instead, they promote the principle that “every move counts” and encourage individuals to replace sedentary behavior with physical activity of any intensity. In addition, a recent global expert consensus led by Yin [32] highlighted that engaging in multiple bouts of short-duration accumulated exercise (SBAE) throughout the day may provide acute benefits for cognitive performance, although the overall quality of the available evidence remains relatively low. At the same time, other studies have reported that sitting interruptions do not consistently produce acute cognitive benefits [33]. This inconsistency suggests that the acute cognitive effects of sitting interruptions, as well as their potential moderating factors, require further clarification through more refined quantitative synthesis. Cognition is not a unitary construct but a set of interrelated mental processes that support the acquisition, maintenance, manipulation, and use of information for goal-directed behaviour [34]. In the present review, cognitive outcomes were therefore conceptualised as domain-specific rather than as a single global outcome. Executive function refers to higher-order control processes that support inhibition, conflict monitoring, cognitive flexibility, task switching, and goal-directed regulation [35, 36]. Memory function refers primarily to the short-term maintenance, updating, or retrieval of information, including working-memory-related task performance when the task primarily requires online retention or updating [37]. Information processing reflects the speed and efficiency with which cognitive or psychomotor information is encoded, recognized, and responded to, whereas attention refers to alertness, sustained attention, or selective allocation of cognitive resources [38, 39]. Global cognition was reserved for composite indices or overall cognitive scores that were explicitly reported as reflecting general cognitive performance [34]. This domain-specific distinction is important because sitting interruptions may plausibly affect prefrontal control processes, memory-related updating, and processing efficiency through partly different acute physiological pathways, and because pooling all cognitive outcomes into a single estimate could obscure domain-specific effects.

A domain-specific pattern of acute cognitive response is theoretically plausible. From a dual-process perspective, cognition involves both relatively automatic, low-effort processing and more effortful, controlled processing that depends on attention allocation, working memory, inhibitory control, and cognitive flexibility [40]. Prolonged uninterrupted sitting may create a physiological and behavioural context characterized by low muscular activity, reduced metabolic demand, lower arousal, and potential declines in cerebral perfusion. These conditions may be particularly relevant to effortful cognitive operations, such as executive control and memory updating, which require sustained prefrontal engagement and are sensitive to fluctuations in arousal, vascular function, and energy availability [41]. By contrast, more automatic or basic processing components may be less responsive to a short bout of standing or light physical activity. This interpretation is also consistent with contemporary movement-behaviour frameworks, which view physical activity, sedentary behaviour, and sleep as interrelated components of a 24-hour behavioural system rather than isolated exposures [42]. Within this framework, sitting interruptions can be understood as short intra-day reallocations from prolonged sedentary time to standing or light-to-moderate physical activity. Such reallocations may influence cognition through both physiological pathways, including cerebral blood flow, glucose regulation, and neurotrophic signalling, and behavioural pathways, including arousal, task engagement, and contextual disruption. Accordingly, the present review assumes that the cognitive effects of sitting interruptions are likely to depend not only on the presence of physical activity, but also on the type of cognitive domain assessed, the nature of the sedentary behaviour being interrupted [43], the timing of cognitive assessment, and the experimental or real-world context in which the interruption occurs.

At present, the evidence synthesis on the effects of sitting interruptions on acute cognitive performance is limited by two major issues. First, most existing reviews synthesise the available quantitative evidence using narrative or qualitative approaches rather than formal meta-analytic methods [44–48]. Given the substantial heterogeneity in intervention modalities, task paradigms, and measurement time points, it remains difficult to generate robust quantitative estimates of acute effects across different cognitive domains. Second, although existing quantitative studies represent an important advance, they still have limitations in both research focus and methodology. For example, Feter et al. [49] conducted a comprehensive review of the effects of increased physical activity and reduced sedentary behavior on cognition, brain function, and brain structure across the lifespan. Although broad in scope, that review did not specifically examine the acute, context-specific strategy of briefly standing or engaging in physical activity to interrupt prolonged sitting, nor did it provide a detailed quantitative synthesis focused on specific cognitive domains and interruption parameters. Understanding acute effects provides information that is complementary to, rather than a substitute for, evidence on lifespan cognitive health. Lifespan studies are essential for evaluating cumulative exposure and long-term cognitive ageing, but they cannot determine whether a short interruption introduced during a single prolonged sitting episode produces an immediate cognitive response. Acute experimental evidence is particularly relevant for workplaces, classrooms, and other settings in which prolonged sitting occurs within a day and where brief, low-cost behavioural changes may be easier to implement than structured exercise programmes. It can also help identify the cognitive domains and interruption parameters that warrant testing in longer-term trials. Therefore, focusing on acute effects allows the present review to address a more proximal and implementation-oriented question: whether sitting interruptions can produce immediate, domain-specific cognitive responses under controlled experimental conditions. By contrast, Li et al. [50] provided the first quantitative evidence on brief physical activity during sedentary periods; however, they used a traditional two-level random-effects model and simplified the analysis by retaining only a single measurement time point and pre-averaging results from multiple tasks within the same cognitive domain into one effect size. Moreover, that study included only nine trials, resulting in a limited evidence base. Although these methodological decisions improved analytical feasibility, they may have reduced the information captured from multiple tasks and time points within individual studies, thereby limiting the ability to detect heterogeneity in acute cognitive effects and their potential determinants.

In view of these limitations, the present study aims not merely to update the existing evidence but to advance the field of evidence synthesis in three respects: research focus, statistical methodology, and clinical interpretation. First, we explicitly restrict our scope to sitting interruptions involving standing or physical activity breaks during prolonged sedentary periods in randomized crossover trials, thereby distinguishing our work from broader studies on general sedentary behavior reduction or physical activity promotion. Second, we apply a three-level model to address effect-size dependency while preserving, as far as possible, the information derived from multiple tasks and time points within individual studies, thereby improving the completeness and robustness of the estimates. Third, we perform stratified analyses across five domains: global cognition, executive function, memory function, information processing, and attention. In addition, through subgroup analyses and meta-regression, we examine the potential influence of participant characteristics, experimental settings, and interruption parameters. Importantly, these exploratory analyses were designed to identify potential sources of heterogeneity and generate hypotheses about possible effect modifiers, rather than to provide confirmatory evidence of dose–response relationships or to determine an optimal prescription for sitting interruptions. Accordingly, the central question of this study is as follows: In which cognitive domains, in which populations, and under which experimental conditions are sitting interruptions most likely to be associated with beneficial acute cognitive effects? By addressing this question, we aim to provide clearer guidance for subsequent high-quality research on underlying mechanisms and clinical translation.

## Methods

This study strictly followed the *Preferred Reporting Items for Systematic Reviews and Meta-Analyses (PRISMA) 2020 Statement* in conducting the three-level meta-analysis. The complete PRISMA 2020 checklist is provided in Supplementary 1. In addition, the study was registered in the International Prospective Register of Systematic Reviews (PROSPERO) under registration number CRD420251273873.

### Information source

We searched PubMed (NCBI), Web of Science (Core Collection), the Cochrane Library, Embase, and Scopus from their inception through January 2026. Studies included in this review were required to be full-text articles written in English and published as peer-reviewed journal papers. No restrictions were placed on publication dates or sample sizes during the search phase.

### Search strategy

Search terms were organized into four categories based on the PICOS framework (Population, Intervention, Comparison, Outcome, and Study Design): Intervention (e.g., “sitting interruptions”), Comparison (e.g., “prolonged sitting”), Outcome (e.g., “cognitive function”), and Study Design (e.g., “randomized crossover trial”). Using Web of Science as an example, the search was conducted within the title, abstract, and keywords fields with the following strategy: TS= (sedentary OR sitting) AND TS= (break* OR interrupt* OR intersperse* OR activity break*) AND TS= (cognition* OR executive function OR attention OR memory OR information processing). This strategy was similar to that used in a previously published systematic review [49], and the full search strategy is provided in Supplementary 2. To maximize the comprehensiveness of study identification, we also implemented three systematic snowballing methods: (i) screening the reference lists of all included studies; (ii) identifying and screening all articles that cited the included studies; and (iii) conducting “related articles” or “similar articles” searches for each included study in MEDLINE and Embase. In addition, we screened relevant articles included in or identified by previous systematic reviews on the effects of sitting interruptions on health outcomes. Finally, we searched PROSPERO and the Cochrane Database of Systematic Reviews to determine whether any relevant systematic review protocols had been registered or published.

### Eligibility criteria

Inclusion and exclusion criteria were defined according to the PICOS framework. Studies were included only if they met all of the following criteria: (i) Participants: human participants of any age and health status; (ii) Intervention: implementation, within a single experimental day characterized by prolonged uninterrupted sedentary behavior, of an interruption protocol comprising at least two discrete interruptions delivered according to a predetermined schedule during the sedentary period, in the form of standing or light-to-moderate-intensity physical activity (e.g., walking); (iii) Comparison: continuous sedentary behavior on the same experimental day without planned interruptions, allowing only necessary brief departures from sitting (e.g., restroom use) when explicitly stated in the original study; (iv) Outcomes: reporting of at least one cognitive outcome, including but not limited to global cognition, executive function, memory function, information processing, and attention, with data available for extraction or conversion into effect sizes; and (v) Study design: randomized crossover trials in which the same participants completed both the continuous sedentary and interrupted sedentary conditions in a randomized order.

Studies were excluded if they met any of the following criteria: (i) the crossover trial did not include a washout period; (ii) uncontrolled exposure to rest or physical activity occurred outside the designated intervention period, potentially resulting in contamination of the intervention or control condition; or (iii) the study was a qualitative study, a free-living intervention or trial, an observational study, a systematic review or meta-analysis, a study protocol, gray literature, or a conference abstract.

### Study selection

EndNote X9 was used to remove duplicate records from the retrieved studies. After deduplication, two researchers independently screened the studies in Zotero (*v7.0.22*) according to the predefined inclusion and exclusion criteria. Any disagreements were resolved through discussion to reach consensus; when consensus could not be achieved, a third researcher was consulted to arbitrate. If the data reported in the included studies were insufficient for in-depth analysis, attempts were made to contact the original authors to obtain additional data.

### Data extraction and data coding

Using a predefined data-extraction form, two researchers independently extracted and cross-checked the data. Any discrepancies were resolved through discussion, with a third researcher serving as an arbiter when necessary. The extracted information included: (i) basic study characteristics (first author, publication year, country/region, and study design); (ii) participant characteristics (sample size, age, sex distribution, body mass index, and status); (iii) characteristics of the intervention and control conditions, including FITT-based interruption characteristics, experimental setting, and washout-period design; (iv) cognitive outcome information (measurement instruments, specific subtests, type of outcome metric [e.g., reaction time, accuracy, or composite scores], measurement time points, baseline and post-intervention values under each condition, or statistics that could be used to calculate effect sizes for paired comparisons); and (v) data required for effect-size calculation (means, standard deviations, standard errors, 95% confidence intervals, *t* values, *P* values, or other convertible statistics).

For data presented only in graphical form, the original authors were first contacted to obtain the raw numerical values. If no response was received and the figure quality permitted, WebPlotDigitizer (*v4.8*) was used to extract the data digitally. Two researchers independently performed the extraction on each figure, and the final value for each data point was taken as the mean of the two extractions when their relative difference was ≤ 5%; otherwise, the figure was jointly re-extracted, with a third senior researcher arbitrating any persisting discrepancies. WebPlotDigitizer has been independently validated for inter-coder reliability and accuracy in extracting graphical data [51]. Raw project files are available from the corresponding author upon reasonable request.

If a study reported the standard error (SE) or 95% confidence interval (CI), these values were converted to standard deviations (SDs) using the formulas recommended in the Cochrane Handbook. A complete audit trail of all data-extraction and conversion procedures was maintained to facilitate verification.

If a study reported a confidence interval (CI), it was converted to a standard deviation (SD) [52]. :

1 $$\:\:\:\:\:\:\:\:\:\:\:\:\:\:\:\:\:\:\:\:\:\:\:\:\:\:\:\:\:\:\:\:\:\:\:\:\:\:\:\:\:\:\:\:\:\:SD=\sqrt{N}\:\frac{{CI}_{high}-\:{CI}_{low}}{2t}\:\:$$

If a study reported the standard error (SE), it was converted to the standard deviation (SD) [52]. :

2 $$\:\:\:\:\:\:\:\:\:\:\:\:\:\:\:\:\:\:\:\:\:\:\:\:\:\:\:\:\:\:\:\:\:\:\:\:\:\:\:\:\:\:\:\:\:\:\:\:\:\:\:SD=\sqrt{N}\:\times\:SE$$

#### Cognitive outcome coding and domain classification

Prior to the formal statistical analysis, we established a priori coding rules for cognitive outcomes and classified all outcomes into five domains: global cognition, executive function, memory function, information processing, and attention. This classification was informed by established multidimensional models of cognition and cognitive assessment, particularly frameworks that distinguish higher-order executive control, working-memory-related processes, information-processing speed, attentional processes, and global cognitive performance [34–39]. For example, executive-function frameworks conceptualize core executive processes as including inhibition, updating or working memory, and cognitive flexibility or shifting, which together support more complex goal-directed behaviours [35, 36]. Working-memory models emphasize the short-term maintenance and manipulation of information [37], whereas processing-speed and attention frameworks emphasize the efficiency of cognitive or psychomotor operations and the selective allocation or maintenance of cognitive resources [38, 39]. In applying these frameworks to the present evidence base, we recognized that many experimental cognitive tasks are process-mixed rather than process-pure. Therefore, each outcome was coded according to the primary cognitive component most directly targeted by the task design and by the interpretation provided in the original study.

If a study reported a global cognitive index, a composite score derived from multiple tasks, or a metric explicitly interpreted by the authors as representing overall cognitive performance, it was coded as global cognition. By contrast, if a study reported a single task or subtask, it was coded according to the primary cognitive component measured by that task. In general, tasks involving inhibitory control, cognitive flexibility, task switching, conflict monitoring, or verbal fluency (e.g., Stroop, Flanker, TMT/TMT-B, task switching, and verbal fluency) were classified as executive function [35, 36]. Tasks primarily reflecting short-term maintenance, online updating, or working-memory load (e.g., 1-back, 2-back, N-back, and Letter Memory) were classified as memory function [37]. Metrics primarily reflecting reaction speed, recognition speed, or psychomotor processing speed were classified as information processing, whereas metrics primarily reflecting alertness, sustained attention, or selective attention were classified as attention [38, 39]. When a task could reasonably be linked to more than one domain, the final classification was based on the dominant process required by the specific outcome metric rather than on the broad task name alone. For example, a reaction-time outcome from a task battery was not automatically coded as executive function unless the metric primarily reflected inhibitory control, switching, or conflict monitoring; similarly, working-memory tasks were coded as memory function when the main requirement was online maintenance or updating of information, while executive-control tasks were coded as executive function when they primarily required inhibition, flexibility, or conflict resolution.

When a single test platform comprised multiple subtasks (e.g., TAP, COMPASS, or similar batteries), the subtasks were assigned to different cognitive domains as appropriate. When a single task reported both reaction time and accuracy, and both metrics represented the same underlying cognitive component, they were extracted separately, coded in a consistent direction, and included as correlated effect sizes within the same study cluster in the three-level model. The complete task-to-domain mapping rules and corresponding measurement instruments are provided in the Supplementary 3.

The categorization of cognitive outcomes into specific domains was performed independently by two researchers. Any disagreements regarding the theoretical classification of a specific task were resolved through discussion, and a third researcher served as an arbiter when necessary.

#### Coding of interruption characteristics

Interruption characteristics were coded according to the FITT framework. Frequency was defined as the rate of interruptions and was expressed as the interval between successive bouts, with bouts per hour calculated where applicable. Intensity was classified as low or moderate according to American College of Sports Medicine (ACSM) criteria, supplemented, where reported, by METs, heart rate, or rating of perceived exertion. Intensity was coded at the experimental-condition level. When a condition included activity breaks of different reported intensities, we assigned the dominant intensity category based on the largest proportion of prescribed interruption exposure; if no single category was dominant, a time-weighted judgment was made using ACSM criteria and available METs, heart rate, or rating of perceived exertion data. When different modalities or intensities were delivered as separate crossover conditions, each condition was coded separately and compared with the prolonged-sitting control condition. Time included the duration of each bout, the total interruption time, the total experimental sedentary-exposure duration, and the timing of cognitive assessment relative to the bouts. Type referred to the modality of the bout, including standing, walking, stair climbing, cycling, resistance exercise, or intermittent jiggling. These coded parameters subsequently served as the basis for the moderator analyses described in Sect. "Subgroup analysis and meta-regression*"*.

### Risk of bias assessment

Studies meeting the inclusion criteria were assessed for risk of bias using the Cochrane RoB 2 tool [53]. This tool evaluates five domains: (i) the randomization process; (ii) deviations from the intended interventions (including the effects of assignment to and adherence to the interventions); (iii) missing outcome data; (iv) measurement of the outcome; and (v) selection of the reported result. In accordance with Cochrane guidance, risk of bias was summarized at the study level [52] as follows: low risk, if all domains were rated as low risk; some concerns, if at least one domain was rated as “some concerns” and no domains were rated as high risk; and high risk, if any domain was rated as high risk. Two reviewers independently performed both the domain-level and overall assessments, and any disagreements were resolved by a third reviewer.

### Statistical analysis

#### Data synthesis

Because this study included only randomized crossover trials, the fundamental unit for effect-size construction was the within-participant paired comparison between the interrupted-sitting condition and the prolonged-sitting condition, rather than a comparison between two independent samples. Accordingly, we prioritized extracting statistics that directly reflected paired differences between the two conditions, including between-condition mean differences, differences in change scores, paired t values, standard errors, 95% confidence intervals, and other convertible statistics. These statistics were used to calculate the standardized mean difference (SMD) and its sampling variance, which were then converted to Hedges’ g with a small-sample correction. If an original study did not directly report paired-comparison results but provided only baseline and post-intervention values for both conditions, we first calculated the change score within each condition (post–pre) and then constructed the paired difference, defined as the change score in the interrupted-sitting condition minus that in the prolonged-sitting condition, as the basis for the effect size.

(a) Correlation used to derive the variance of within-participant paired comparisons (*r* = 0.5). When a study did not report the standard deviation (or standard error) of this paired difference directly, we reconstructed it from the condition-level standard deviations, assuming a conservative correlation of *r* = 0.5 in the primary analysis [54]. This correlation therefore serves a single, local purpose, namely estimating the sampling variance of one paired effect size, and its influence was examined in sensitivity analyses using *r* = 0.3 and *r* = 0.7 (Supplementary 8).

To avoid interpretive confusion arising from inconsistent outcome directions, all effect sizes were harmonized before pooling: a positive value indicated better cognitive performance under the interrupted-sitting condition than under the prolonged-sitting condition. Accordingly, for outcomes in which lower values indicated better performance (e.g., reaction time), the direction was reversed before calculation. For studies reporting multiple cognitive tasks, subtasks, or measurement time points, each was retained as a separate effect-size entry; however, the statistical dependency among these entries was addressed in the subsequent models by treating study as the clustering unit. For studies that compared more than one interruption arm with a single prolonged-sitting control condition, each interruption-versus-control contrast was computed as a separate effect size. The shared control sample was not divided across intervention arms. Instead, dependency among contrasts from the same study was handled within the multilevel modelling framework described below: all effect sizes from a given study were retained within a single Level-3 study cluster, residual variation among multiple effect sizes from the same study was captured at Level 2, and the off-diagonal elements of the sampling variance–covariance matrix represented the assumed sampling covariance among effect sizes sharing the same study cluster, including dependency arising from the shared control condition.

#### Three-level meta-analysis

Research on cognitive function typically employs a wide range of measurement tools, such as the Stroop test, N-back task, Flanker test, and Trail Making Test (TMT) [20]. In addition, repeated measurements across multiple cognitive domains and time points may be obtained within a single experimental session [55]. Consequently, a single study often yields multiple mutually dependent effect sizes. Treating these effect sizes as independent would violate the independence assumption of traditional meta-analysis and could lead to underestimation of standard errors [56]. Conversely, selecting only one outcome or retaining only a single effect size may be overly conservative and may fail to capture the underlying effect adequately [57].

To accommodate the statistical dependency arising from multiple effect sizes reported within the same study, we fitted three-level random-effects models that partition the total variance of the observed effect sizes into three hierarchical components [58, 59]. Level 1 represents the sampling variance of each individual effect size (the within-participant estimation error of each Hedges’ g, treated as known and fixed at its estimated value). Level 2 represents the variance among the multiple effect sizes nested within the same study (σ²_within; e.g., different cognitive tasks, outcome metrics, or measurement time points obtained from the same participants). Level 3 represents the variance between studies (σ²_between). To illustrate this structure, Heiland et al. [60] contributed multiple memory-related effect sizes from N-back outcomes under both walking and resistance-exercise interruption conditions in the same participants. In this example, the sampling variance of each individual Hedges’ g estimate was represented at Level 1; the residual variation among multiple effect sizes derived from the same study was captured at Level 2 (σ²_within); and the variation between Heiland et al. [60] and the other included studies was captured at Level 3 (σ²_between).

Models were fitted in R (*v4.4.3*) with the *metafor* package [61] using rma.mv(yi, V, random = ~ 1 | study / esid), where yi is the vector of Hedges’ g values, V is the variance–covariance matrix of the sampling errors, with the diagonal elements representing the sampling variances of individual paired effect sizes and the off-diagonal elements representing the assumed covariance among multiple effect sizes from the same study, study indexes the Level-3 clustering unit, and esid indexes individual effect sizes nested within studies (Level 2). The two random-effects variance components (σ²_within and σ²_between) were estimated by restricted maximum likelihood (REML), with maximum likelihood (ML) used as a robustness check [62]. The contribution of each level was quantified with a multilevel I² (the proportion of total variance attributable to Level 2 and to Level 3), and the statistical significance of each component was examined with likelihood-ratio tests comparing the full model against reduced models in which σ²_within or σ²_between was constrained to zero [59]. Because effect sizes were nested within studies and the assumed working covariance structure (described in paragraph (b) below) may be mis-specified, we re-estimated all standard errors using cluster-robust (sandwich) variance estimation (RVE) with the CR2 small-sample correction and Satterthwaite degrees of freedom [63, 64]. The clustering unit was the individual study: each primary publication formed one cluster, so that all effect sizes from a study, including those from multiple interruption arms, multiple cognitive tasks, and multiple measurement time points, were treated as a single cluster. Following standard recommendations, coefficients whose Satterthwaite degrees of freedom fell below 4 were regarded as unreliable and were not interpreted.

(b) Correlation used to model dependency among multiple effect sizes from the same study (*r* = 0.6). A separate and conceptually distinct assumption was required because several studies contributed more than one effect size to the same analysis (e.g., multiple cognitive tasks or metrics measured in the same participants). To account for the resulting statistical dependency, we constructed the variance–covariance matrix V of the sampling errors with an assumed constant correlation of *r* = 0.6 among effect sizes sharing the same study cluster [65]. This correlation governs the off-diagonal (covariance) elements of V used by the multilevel model; it does not affect the variance of any single paired comparison. Its influence was examined in sensitivity analyses using *r* = 0.4 and *r* = 0.8 (Supplementary 8).

Effect sizes were expressed as standardized mean differences (SMDs), with Hedges’ g used to correct for small-sample bias. Effect sizes were interpreted as trivial (*g* < 0.2), small (0.2 ≤ *g* < 0.5), medium (0.5 ≤ *g* < 0.8), or large (*g* ≥ 0.8) [66]. To ensure directional consistency across cognitive task metrics, such as outcomes for which shorter reaction times indicate better performance and outcomes for which higher accuracy indicates better performance, all effect sizes were harmonized before pooling so that positive Hedges’ g values consistently indicated better cognitive performance. Forest plots were generated using the orchard package (*v2.0*).

In addition to reporting the 95% confidence intervals (CIs) for the pooled effects, this study also calculated prediction intervals (PIs) for the primary outcomes based on the t distribution [67]. This approach was adopted because, within a random-effects framework, PIs reflect the range of true effects that may arise from between-study heterogeneity and thus provide information supplementary to that offered by CIs [68, 69]. Heterogeneity was assessed using Cochran’s Q, I², and the variance component (*τ²*). Furthermore, within the three-level framework, both within-study (Level 2) and between-study (Level 3) variance components, together with their relative contributions, were reported to provide a more comprehensive characterization of heterogeneity sources [59]. In addition, as a supplementary analysis, we used the *metameta* package to calculate the statistical power of the primary pooled effect sizes. This analysis was intended to assess the risk of false-negative findings given the observed sample sizes and heterogeneity levels, although the results were interpreted only as supportive evidence [70].

#### Subgroup analysis and meta-regression

To explore sources of heterogeneity and generate hypotheses for future research, this study prospectively planned subgroup and meta-regression analyses [71]. Given the limited number of included studies, with some subgroups containing only a few studies or even a single study, and the presence of multiple correlated effect sizes within individual studies, all subgroup and meta-regression analyses were considered exploratory. Their primary purpose was to characterize potential directions of effect modification and identify leads for further validation, rather than to provide confirmatory evidence or serve as a direct basis for determining optimal prescription parameters.

Subgroup analyses were conducted to examine differences in effect-size distributions across categorical moderators, with interruption-related variables organised according to the FITT framework where applicable. The prespecified categorical moderators included age group (children: 4–9 years; adolescents: 10–17 years; young adults: 18–35 years; middle-aged adults: 36–64 years; older adults: ≥65 years) [72, 73], BMI category (normal weight: 18.5–24.9 kg/m²; overweight/obesity: ≥25 kg/m²), experimental setting, interruption type, and interruption intensity. Interruption type and intensity corresponded to the Type and Intensity components of the FITT framework, respectively; intensity was classified as low or moderate according to ACSM recommendations [74].

Meta-regression analyses were conducted for continuous moderators, with FITT-related variables organized as Frequency and Time parameters. The prespecified continuous moderators included age, BMI, sex distribution expressed as the percentage of female participants, interruption frequency expressed as inter-bout interval, duration of each interruption, total number of interruptions, and total experimental sedentary-exposure duration per day. Interruption frequency corresponded to the Frequency component, whereas duration-related variables corresponded to the Time component. All regression coefficients and associated *p* values were interpreted as exploratory and hypothesis-generating rather than as evidence of causal or dose-response relationships. Meta-regression results were visualized using the *ggplot2* package [75].

#### Publication bias and sensitivity analysis

Publication bias and small-study effects were assessed primarily through visual inspection of funnel plots combined with Egger’s regression test [76, 77]. Given that Egger’s test has limited statistical power and unstable performance when the number of independent studies is small and considering that the present study included randomized crossover trials while retaining multiple correlated effect sizes within the same study, a formal Egger test was performed only when a specific outcome included at least 10 independent studies (or clusters of independent studies) [78]. For outcomes with fewer independent studies, only descriptive visual inspection of the funnel plot was conducted, and no formal statistical judgment regarding publication bias was made on the basis of visual inspection alone.

It should be emphasized that, given the data structure of the present study, funnel-plot asymmetry and the results of Egger’s regression test are best interpreted as indicators of potential small-study effects or signals of funnel-plot asymmetry, rather than as definitive evidence of publication bias. Accordingly, interpretation was based on a comprehensive evaluation of visual inspection, the number of independent studies, the dependency structure of effect sizes, and the contextual evidence for each outcome, rather than on the statistical significance of any single test alone.

Sensitivity analyses included: (i) varying the paired-difference correlation used to reconstruct the variance of within-participant paired comparisons (*r* = 0.3 and *r* = 0.7; see Sect. “Data synthesis”, paragraph (a)); (ii) varying the within-study dependency correlation used to impute the off-diagonal elements of the sampling variance–covariance matrix (*r* = 0.4 and *r* = 0.8; see Sect. “Three-level meta-analysis*”*, paragraph (b)); (iii) leave-one-out analyses; (iv) identifying and excluding outliers on the basis of influence diagnostics, specifically when Cook’s distance or leverage values exceeded three times their respective means, or when the absolute value of the studentized residual exceeded 3 [79, 80]. Outlier exclusion was used to assess the robustness of both the overall model and the exploratory moderator analyses, whereas the remaining sensitivity analyses focused primarily on the main pooled effects.

### Certainty of the evidence

Two researchers independently assessed the certainty of evidence for each major outcome using the Grading of Recommendations Assessment, Development, and Evaluation (GRADE) framework, classifying the evidence into four levels: high, moderate, low, or very low [81]. Because all studies included in this review were randomized crossover trials, the initial certainty rating was set to high. The evidence was then evaluated outcome by outcome and downgraded, where appropriate, according to the five GRADE domains: risk of bias, inconsistency, indirectness, imprecision, and publication bias. Any disagreements between the two researchers were resolved through discussion, and a third researcher served as an arbiter when necessary.

For risk of bias, we used the RoB 2 tool to make an overall judgment at the outcome level across the included studies. This judgment considered the proportion of studies rated as low risk, some concerns, or high risk for a given outcome, as well as the likely influence of these ratings on the main conclusions, rather than relying on automatic downgrading based solely on the rating of any single study. For inconsistency, we considered the consistency of effect directions, statistical heterogeneity indices (e.g., *I*², Cochran’s Q, and variance components in the three-level model), whether the prediction intervals crossed the null, and whether differences in study design, task paradigms, or population characteristics could account for heterogeneity. Evidence was therefore not downgraded mechanically based on fixed I² thresholds alone. For indirectness, we assessed whether the populations, interventions, comparators, and cognitive outcomes examined in the included studies directly matched the review question. When the primary evidence was derived mainly from specific age groups, laboratory settings, or acute-exposure designs, or when discrepancies existed between the measured outcomes and the target constructs, downgrading was considered as appropriate. For imprecision, judgments were based on the width of the 95% confidence intervals around the pooled effects, whether those intervals crossed the null, the range of the prediction intervals, the adequacy of the sample size and number of independent studies, and the stability of the point estimates; statistical non-significance alone was not considered sufficient grounds for downgrading. For publication bias, we made a comprehensive judgment based on the number of independent studies, funnel-plot symmetry, the results of Egger’s test when applicable, and the likelihood of small-study effects; no automatic downgrade was applied solely based on the p value from Egger’s test. Ultimately, the certainty of evidence for each outcome was determined through an overall assessment of these five domains. A GRADE evidence profile table is provided in the Supplementary 10 to document the rationale for downgrading and the final rating assigned to each outcome.

## Results

### Search results

**Fig. 1:**
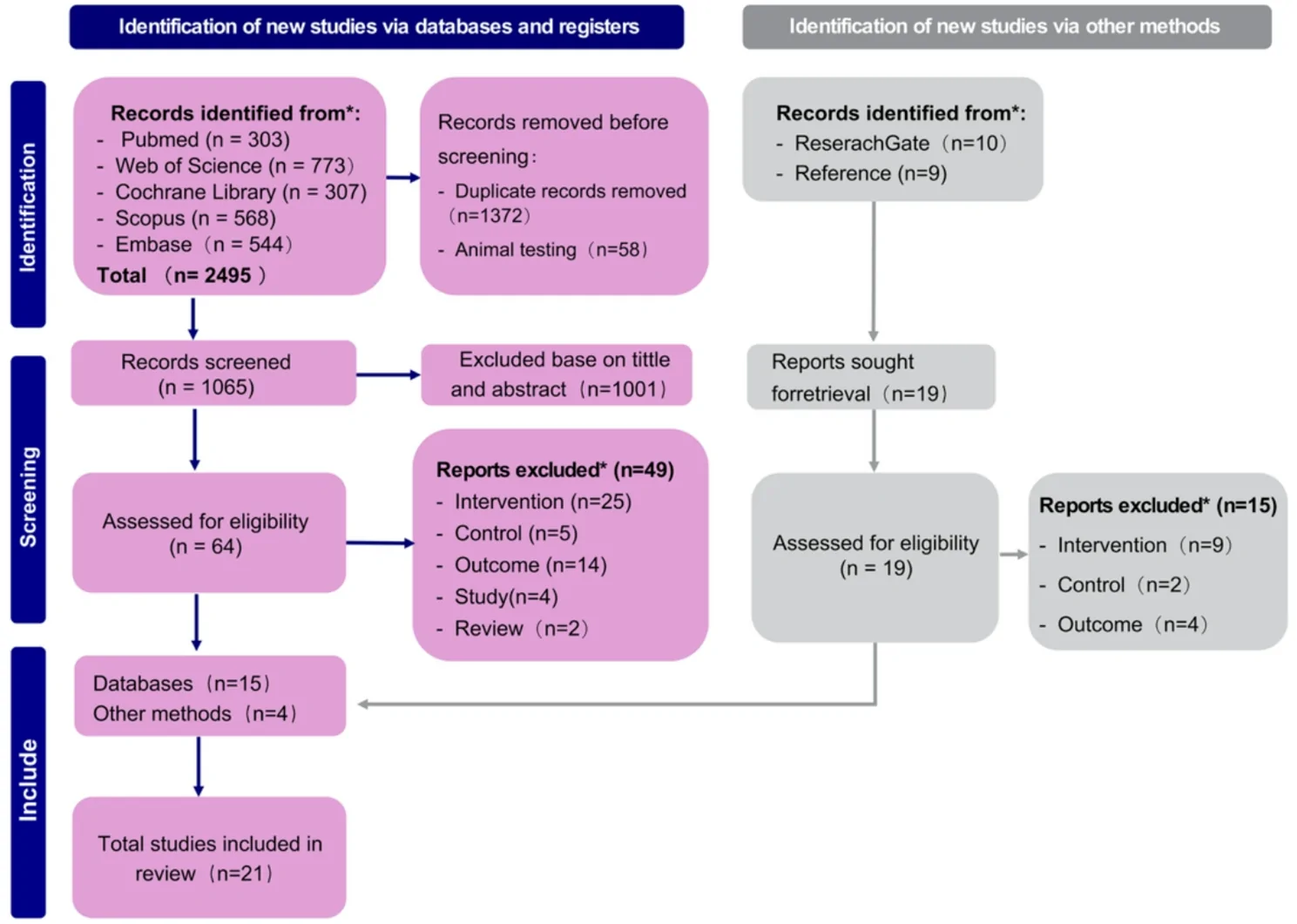
PRISMA flow diagram of study selection

As shown in Fig. 1, the database search identified a total of 2495 records (PubMed, *n* = 303; Web of Science, *n* = 773; Cochrane Library, *n* = 307; Scopus, *n* = 568; and embase, *n* = 544). Before the initial screening, 1430 records were removed, leaving 1065 records for title and abstract screening. Of these, 1001 were excluded, and 64 proceeded to full-text review. During full-text screening, 47 articles were excluded, resulting in the inclusion of 17 studies identified through database searches. An additional 19 records were identified from other sources (ResearchGate, *n* = 10; Reference lists, *n* = 9). After full-text assessment, 15 of these records were excluded, resulting in the inclusion of 4 additional studies. In total, 21 studies were included in this systematic review and meta-analysis.

### Characteristics of included studies

All 21 included studies were randomized crossover trials [60, 82–101], involving a total of 433 participants, 220 (50.8%) of whom were female. Sample sizes ranged from 9 to 67 participants, and participant ages ranged from 13 to 69 years. Six studies [82, 85, 90, 96, 98, 99] enrolled participants with BMI-defined overweight or obesity, whereas the remaining 15 studies [60, 83, 84, 86–89, 91–95, 97, 100, 101] did not specifically select participants according to BMI category. Regarding cognitive outcomes, two studies [82, 85] (9.5%) assessed global cognition; 16 studies [82, 83, 85–96, 99, 100] (76.2%) assessed executive function; 10 studies [60, 82, 84, 85, 90, 93, 96–98, 101] (47.6%) assessed memory function; four studies [85, 87, 90, 97] (19.1%) assessed information processing; and five studies [87, 90, 93, 96, 97] (23.8%) assessed attention. With respect to interruption protocols, walking was used in 14 studies [60, 82, 83, 85–91, 93, 95, 98, 99] (66.7%); standing in two studies [84, 85] (9.5%); resistance exercise in six studies [60, 83, 92, 94, 97, 101] (28.6%); cycling in two studies [85, 96] (9.5%); stair climbing in two studies [95, 101] (9.5%); and jiggling in one study [100] (4.8%). The most common interruption frequency was once every 30 min (11 studies [60, 82, 83, 87–90, 96–99], 52.4%), followed by once every 20 min (3 studies [84, 86, 94], 14.3%) and once every 60 min (3 studies [85, 91, 95], 14.3%). In addition, one study each used intervals of 10 min [92], 25 min [93], 5 min [95], and 17 min [101] (4.8% each). The duration of controlled sedentary time ranged from 80 to 500 min, whereas the duration of a single interruption ranged from 0.5 to 15 min. Further details on participant characteristics and interruption protocols are presented in Tables 1 and 2. Four of the 21 included studies [85, 86, 90, 93] (19.0%) reported the relevant cognitive outcomes only in graphical form; numerical values were extracted using WebPlotDigitizer following the dual-independent-extraction protocol described in Sect. “Data extraction and data coding”.

**Table 1:** Characteristics of included studies and participants

Author (Year)	Country	Participants (*n*)	Sex	Age(years)	BMI (kg/m²)	Status	Outcome	
							Measurement	Domains
Wennberg et al., (2016) [77]	Australia	19	10 M,9 F	59.7 ± 8.1	31.5 ± 4.7	Overweight/Obesity	Flanker、MSCWT、N-back、letter memory	①②③
Silva et al., (2024) [78]	Brazil	12	4 M,8 F	28 ± 9	25.1 ± 4.9	Healthy	Stroop、Flanker	②
Penning et al., (2017) [79]	Australia	18	11 M,7 F	13.5 ± 0.9	21.1 ± 3.9	Healthy	FIT	③
Mullane et al., (2017) [80]	USA	9	7 M,2 F	30 ± 15	29 ± 3	Overweight/Obesity	DT、One -Back、SST	①②③④
Kuo et al., (2024) [81]	Taiwan	24	13 M,11 F	31 ± 8	22.7 ± 2.3	Healthy	TS、Flanker	②
Chrismas et al., (2019) [82]	Qatar	11	11 F	29.75 ± 7.22	23.03 ± 4.90	Healthy	COMPASS	②④⑤
Chueh et al., (2025) [83]	Taiwan	18	18 M	25 ± 4	23.5 ± 3.2	Healthy	TMT、VFT	②
Cunha et al., (2025) [84]	Brazil	27	2 M,25 F	69.4 ± 5.6	25.1 ± 4.6	Healthy	TMT、VFT	②
Wheeler et al., (2020) [85]	Australia	67	35 M,32 F	67 ± 7	31.2 ± 4.1	Overweight/Obesity	GMLT、DT、IDN、OCLT、N-Back	②③④⑤
Bergouignan et al., (2016) [86]	USA	30	9 M,21 F	30 ± 5.6	23.0 ± 2.4	Healthy	CTMT、EFT	②
Stoner et al., (2019) [87]	USA	20	6 M,14 F	21.7 ± 2.5	25.5 ± 6.1	Healthy	Stroop	②
Wu et al., (2023) [88]	The Netherlands	21	6 M,15 F	24 ± 3	23 ± 2	Healthy	D2T、Stroop、TMT、N-Back	②③⑤
Horiuchi et al., (2023) [89]	Japan	20	11 M,9 F	21 ± 1	21.6 ± 1.6	Healthy	Stroop、TMT-B	②
Chandran et al., (2023) [90]	India	21	17 M,4 F	26.65 ± 2.64	22.32 ± 2.46	Healthy	EFT	②
Heiland et al., (2021) [55]	Sweden	13	8 M,5 F	50.5 ± 4.6	24 ± 2.4	Healthy	N-Back	③
Wanders et al., (2021) [91]	The Netherlands	24	5 M,19 F	59.6 ± 8.1	30.2 ± 2.5	Overweight/Obesity	TAP 2.3	②③⑤
Charlett et al., (2021) [92]	UK	12	5 M,7 F	25 ± 6	24.7 ± 4.9	Healthy	PECP	③④⑤
Pindus et al., (2025) [93]	USA	19	7 M,12 F	29.7 ± 7.35	30.0 ± 3.64	Overweight/Obesity	N-back	③
Kuang et al., (2025) [94]	USA	19	7 M,12 F	29.7 ± 7.35	30.0 ± 3.64	Overweight/Obesity	MEFT	②
Fryer et al., (2022) [95]	UK	13	13 M	21.4 ± 1.7	23.9 ± 2.5	Healthy	TMT	②
Kjellenberg et al., (2024) [96]	Sweden	17	6 M,11 F	13.6 ± 0.7	18.9 ± 2.1	Healthy	N-Back	③

Cognitive outcomes were classified into five domains based on the primary cognitive component reflected by each task, with classification rules detailed in Sect. “Cognitive outcome coding and domain classification” and Supplementary 3. Domains:① Global Cognition; ② Executive Function; ③ Memory Function; ④ Information Processing; ⑤ Attention

*Abbreviations *of cognitive tasks: *MSCWT *Modified Stroop Colour-Word Task, *FIT *Figural Intersections Task, *SST *Set-Shifting Test, *TS *Task Switching, *COMPASS *Computerised Mental Performance Assessment System, *TMT *Trail Making Test, *TMT-B *Trail Making Test Part B, *VFT *Verbal Fluency Task(s), *GMLT *Groton Maze Learning Test, *IDN *Identification Task, *OCLT *One Card Learning Task, *CTMT *Comprehensive Trail Making Test, *EFT *Eriksen Flanker Task, *MEFT *Modified Eriksen Flanker Task, *D2T *D2 Attention Test, *TAP *Test of Attentional Performance, *TAP 2.3 *Test of Attentional Performance version 2.3, *PECP *Psychomotor Evaluation Computer Programme, *DT *Detection Test, *N-back *N-back task, *One-Back *One-Back Task, *LMT *Letter Memory Task, *Stroop *Stroop Task/Test, *Flanker *Flanker Task/Test

**Table 2:** Intervention characteristics

Study	Group (*n*)	Setting	Interruption Parameters					
			Type	Intensity	Count (*n*)	Break duration(min)	Frequency(min)	Total duration(min)
Wennberg et al., (2016) [77]	Exp (19)	Laboratory	Walking	3.2 km/h [Low]	10	3	30	420
Silva et al., (2024) [78]	Exp (12)	Laboratory	Resistance	30% MVC [Low]	12	2	30	180
	Exp2 (12)	Laboratory	Walking	Low-intensity walking [Low]	12	2	30	180
Penning et al., (2017) [79]	Exp (17)	Simulated	Standing	Standing only [Low]	9	4	20	370
Mullane et al., (2017) [80]	Exp1(9)	Simulated	Standing	Standing only [Low]	8	15	60	480
	Exp2 (9)	Simulated	Cycling	25–30 rpm [Low]	8	15	60	480
	Exp3 (9)	Simulated	Walking	1.6 km/h [Low]	8	15	60	480
Kuo et al., (2024) [81]	Exp (24)	Laboratory	Walking	6.4 km/h [Moderate]	15	2	20	320
Chrismas et al., (2019) [82]	Exp (11)	Laboratory	Walking	6.0 km/h [Moderate]	9	3	30	300
Chueh et al., (2025) [83]	Exp (18)	Laboratory	Walking	6.4 km/h [Moderate]	7	3	30	210
Cunha et al., (2025) [84]	Exp (27)	Laboratory	Walking	Low-intensity walking [Low]	6	2	30	180
Wheeler et al., (2020) [85]	Exp (67)	Laboratory	Walking	Low-intensity walking [Low]	13	3	30	480
Bergouignan et al., (2016) [86]	Exp (30)	Laboratory	Walking	Moderate-intensity walking [Moderate]	6	5	60	500
Stoner et al., (2019) [87]	Exp (20)	Laboratory	Resistance	Resistance [Low]	17	0.5	10	180
Wu et al., (2023) [88]	Exp (21)	Laboratory	Walking	3.6 km/h [Low]	8	5	25	240
Horiuchi et al., (2023) [89]	Exp (20)	Laboratory	Resistance	Resistance [Low]	8	1	20	180
Chandran et al., (2023) [90]	Exp1 (21)	Laboratory	Walking	Borg RPE 11/20 [Low]	4	3	60	240
	Exp2 (21)	Laboratory	Stair climbing	Borg RPE 13/20 [Moderate]	4	3	60	240
Heiland et al., (2021) [55]	Exp1 (13)	Laboratory	Walking	75–80% HRmax [Moderate]	6	3	30	180
	Exp2 (13)	Laboratory	Resistance	Resistance exercise [Low]	6	3	30	180
Wanders et al., (2021) [91]	Exp1 (24)	Laboratory	Cycling	50–70% HRmax [Moderate]	6	5	30	240
	Exp2 (24)	Laboratory	Cycling	50–70% HRmax [Moderate]	6	5	30	240
Charlett et al., (2021) [92]	Exp (12)	Laboratory	Resistance	Bodyweight resistance [Low]	9	3	30	300
Pindus et al., (2025) [93]	Exp (19)	Laboratory	Walking	55% HRR [Moderate]	5	3.5	30	180
Kuang et al., (2025) [94]	Exp (19)	Laboratory	Walking	55% HRR [Moderate]	5	3.5	30	180
Fryer et al., (2022) [95]	Exp (13)	Laboratory	Jiggling	Jiggling [Low]	36	1	5	180
Kjellenberg et al., (2024) [96]	Exp1(17)	Simulated	Resistance	Resistance exercise [Low]	4	3	17	80
	Exp2 (17)	Simulated	Stair climbing	Stair climbing [Moderate]	4	3	17	80

All 21 included studies were randomized crossover trials in which the same participants completed both conditions on separate experimental days. The control condition was prolonged uninterrupted sitting, with total duration matched to that of the corresponding experimental condition; this homogeneity was guaranteed by the eligibility criteria (see “Eligibility Criteria”). Exercise intensity was uniformly classified as Low or Moderate according to the recommendations of the American College of Sports Medicine (ACSM) and is shown in square brackets at the end of each entry in the Intensity column. Where the original study reported a quantitative metric (e.g., walking speed, %HRmax, %HRR, %MVC, RPE), this metric is presented in the Intensity column; otherwise, the qualitative description used by the original authors is retained

*Abbreviations*: *Exp *Experimental group, *MVC *Maximum Voluntary Contraction, *RPE *Rating of Perceived Exertion, *HRmax *Maximum Heart Rate, *HRR *Heart Rate Reserve, *rpm *revolutions per minute

### Risk of bias assessment

**Fig. 2:**
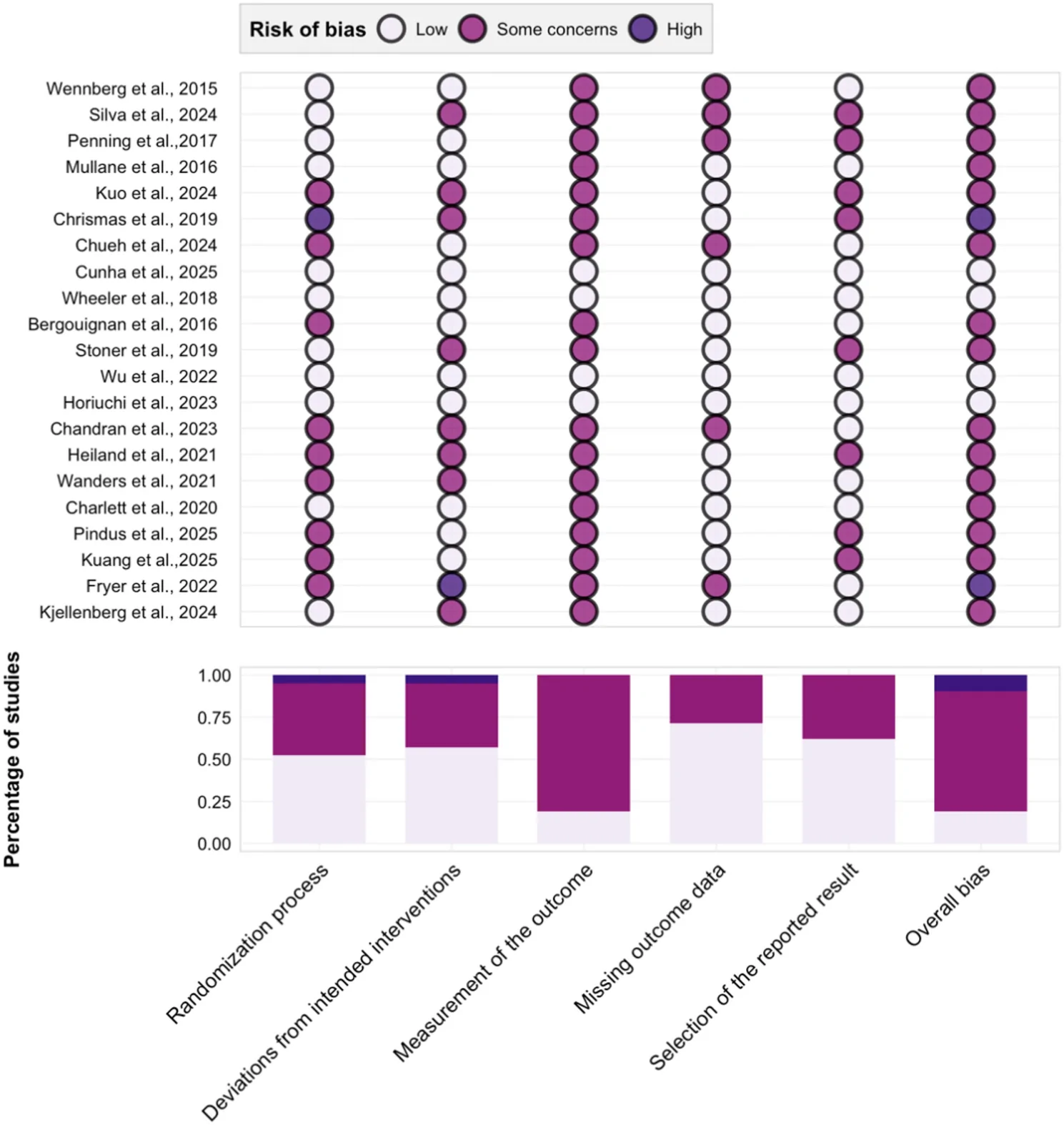
Risk of bias assessment of included studies

Assessment of the 21 randomized crossover trials using the RoB 2 tool indicated that the overall risk of bias was predominantly rated as “some concerns.” Specifically, 4 studies were judged to be at low risk of bias [89, 90, 93, 94], 15 were judged to have some concerns [60, 82–86, 88, 91, 92, 95–99, 101], and 2 were judged to be at high risk of bias [87, 100]. Methodological limitations in the included studies were mainly related to insufficient blinding in outcome assessment, the absence of device-based outcome measurement in some cases, and inadequate reporting of randomization procedures, which contributed to the overall classification of “some concerns” (Fig. 2).

### Meta-analysis and exploratory moderator analyses

This study performed three-level meta-analyses across five prespecified cognitive domains, followed by exploratory moderator analyses. Overall, executive function and memory function showed positive effects, whereas no consistent evidence of improvement was observed for global cognition, information processing, or attention.

#### Global cognition

The evidence base for global cognition remains limited (Supplementary 5). Only two studies [82, 85] contributed a total of eight effect sizes. A three-level meta-analysis showed that, compared with continuous sitting, the pooled effect size for global cognition following brief standing or physical activity breaks during sedentary periods was Hedges’ *g* = 0.57 (95% *CI*: −0.20 to 1.34; *p* = 0.122). Heterogeneity decomposition indicated variability at both the within-study and between-study levels (*I*²_Level 2 = 38.36%; *I*²_Level 3 = 28.21%). In addition, the prediction interval was wide and crossed the null (*PI*: −0.82 to 1.96), suggesting that the current estimate is unstable. Given the very low certainty of evidence, the findings should be interpreted cautiously: current evidence regarding global cognition remains sparse and uncertain and is insufficient to support a definitive conclusion. Because of the limited number of studies, no exploratory subgroup analyses or meta-regression analyses were conducted for this outcome.

#### Executive function

**Fig. 3:**
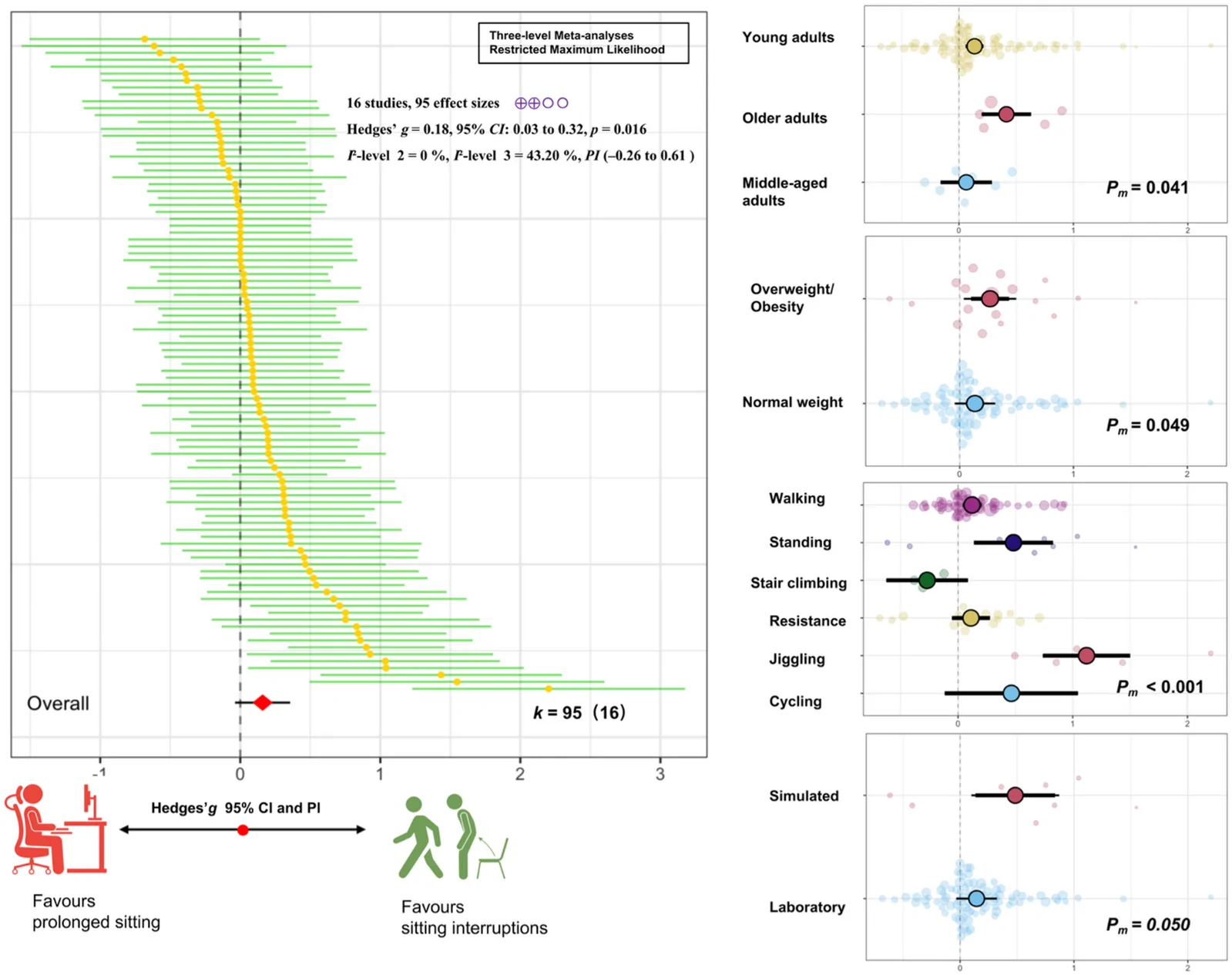
Overall and subgroup effects of sitting interruptions on Executive Function. NOTE: In the left panel, each green horizontal line represents the 95% CI for an individual effect size, and the yellow point indicates the corresponding Hedges’ g estimate. The red diamond indicates the overall pooled effect, and the black horizontal line indicates the 95% prediction interval (PI). In the right-hand subgroup panels, small points represent individual effect sizes, whereas larger points with black horizontal lines represent subgroup estimates and their 95% CIs. Values to the left of zero favour prolonged sitting, whereas values to the right of zero favour sitting interruptions

Executive function was one of the most robustly supported outcomes in this study, encompassing 16 studies and 95 effect sizes (Fig. 3). A three-level meta-analysis showed that, compared with continuous sedentary behavior, brief standing or physical activity breaks during sedentary periods were associated with a pooled effect size for executive function of Hedges’ *g* = 0.18 (95% *CI*: 0.03 to 0.32; *p* = 0.016), indicating a small positive effect. Heterogeneity decomposition indicated no heterogeneity at the within-study level (*I*²_Level 2 = 0%) and moderate heterogeneity at the between-study level (*I*²_Level 3 = 43.20%). In addition, the prediction interval was relatively wide and crossed the null (*PI*: −0.26 to 0.61), suggesting that the magnitude of the effect may vary across study contexts. Given the low certainty of evidence, this finding is best interpreted cautiously: interruptions to prolonged sitting may produce a small acute improvement in executive function, but the consistency and generalizability of this effect remain uncertain.

Exploratory subgroup analyses further suggested that age group (*p* = 0.041), BMI (*p* = 0.049), interruption type (*p* < 0.001), and experimental setting (*p* = 0.050) may be associated with variation in effect size. Descriptive results indicated that point estimates were relatively larger in older adults and in participants with overweight or obesity. Walking showed a relatively consistent positive signal, whereas standing (Hedges’ *g* = 0.48) and jiggling (Hedges’ *g* = 1.12), despite their large point estimates, were each supported by only a single study and should therefore be interpreted with caution.

**Fig. 4:**
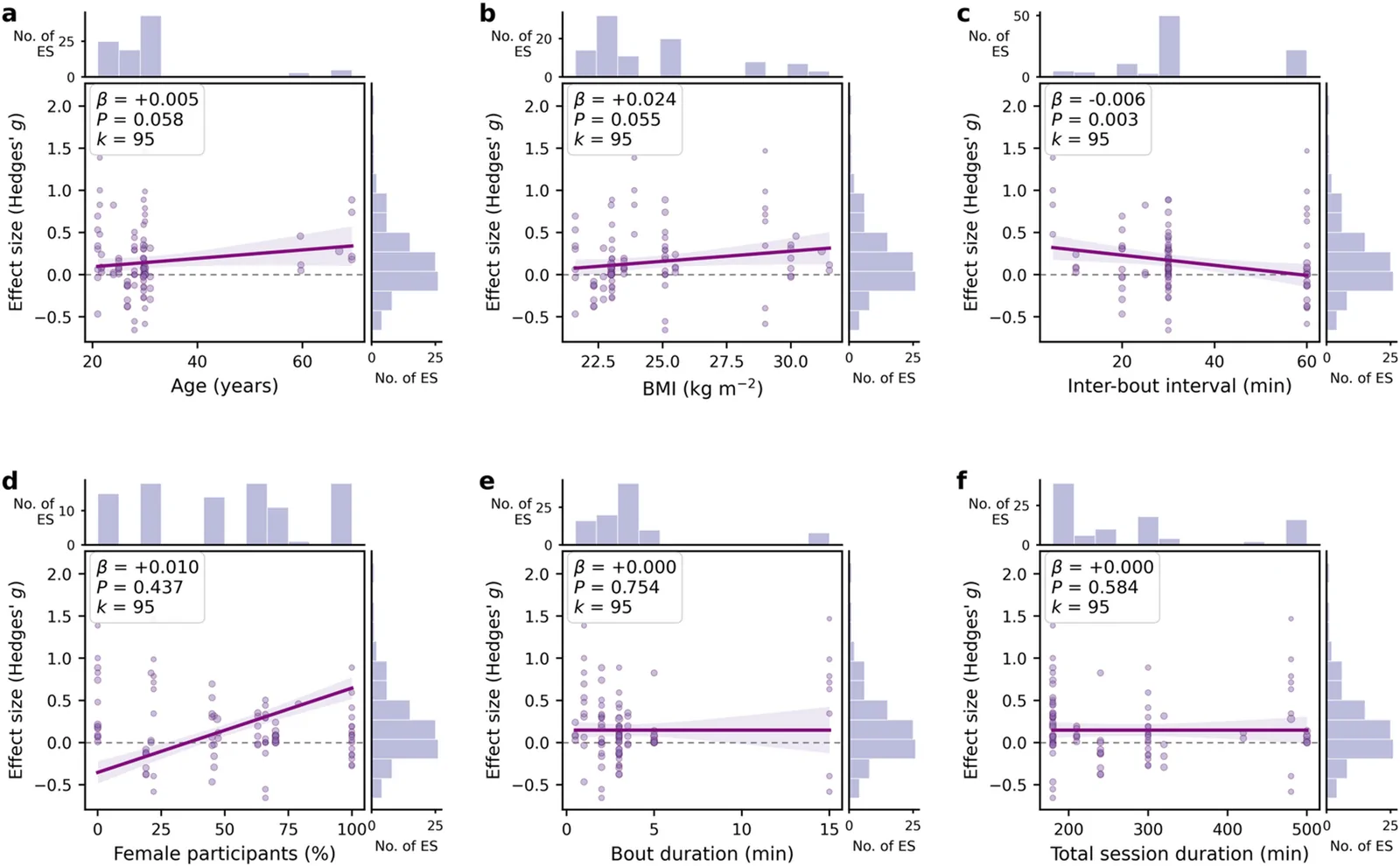
Meta-regression analyses of moderators of Executive Function. Panels represent: **a** age; **b** BMI; **c** inter-bout interval; **d** percentage of female participants; **e** bout duration; and **f** total experimental session duration. Each point represents one effect size. The solid purple line indicates the fitted meta-regression line, and the shaded band represents the 95% confidence interval. The horizontal dashed line marks the null effect (g = 0). Marginal histograms show the distributions of moderator values along the top axis and effect sizes along the right axis, expressed as the number of effect sizes (No. of ES). Each panel reports the regression coefficient (β), P value, and number of effect sizes (k)

Exploratory meta-regression further suggested a negative association between interruption frequency and effect size for executive function (*β* = −0.006, *p* = 0.003), implying that more frequent sitting interruptions may be associated with greater improvements in executive function. Age (*β* = 0.005, *p* = 0.058) and BMI (*β* = 0.024, *p* = 0.055) showed only marginal trends. Neither the duration of a single interruption, total experimental exposure duration, nor sex distribution showed a clear association (Fig. 4).

#### Memory function

**Fig. 5:**
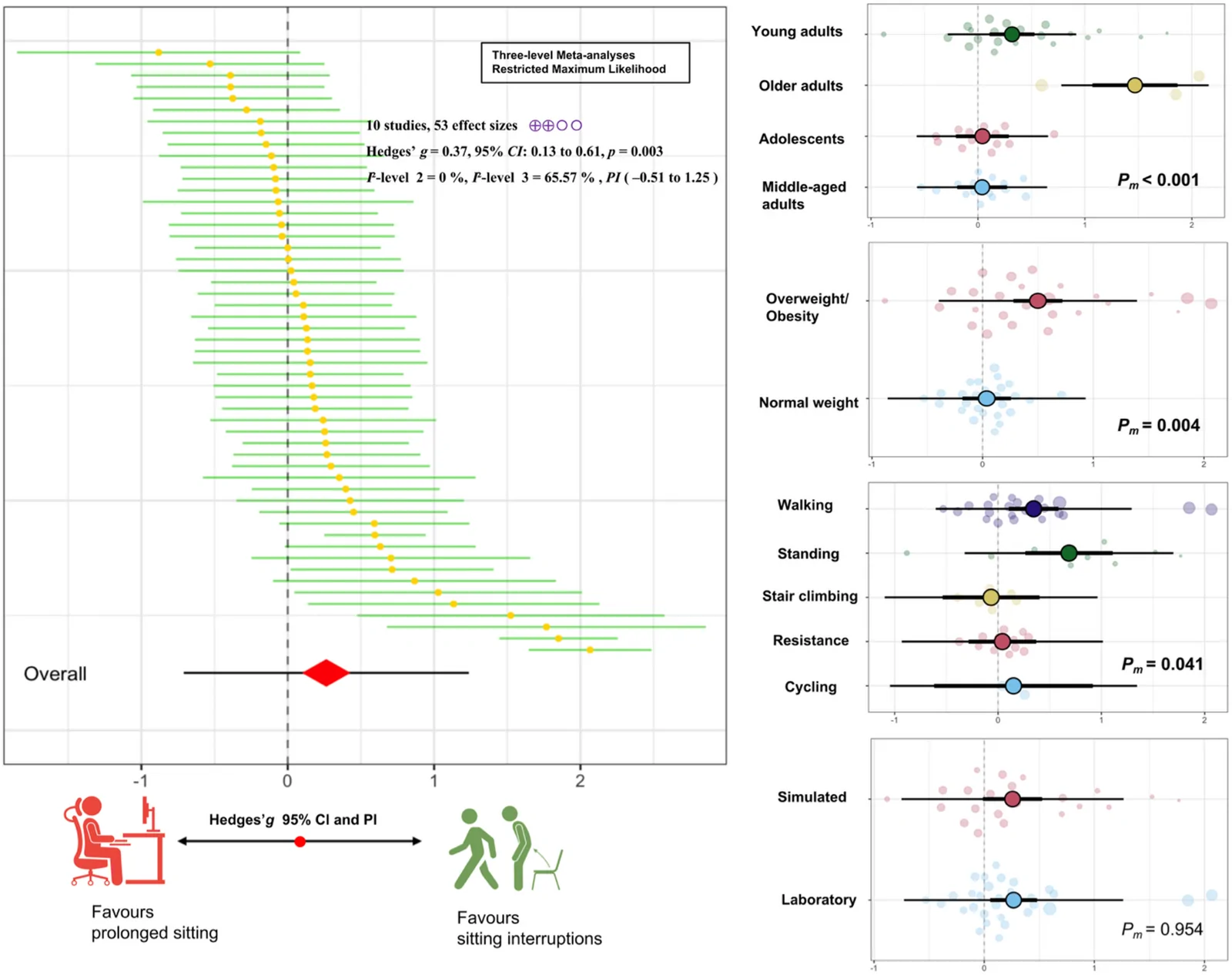
Overall and subgroup effects of sitting interruptions on Memory Function. NOTE: In the left panel, each green horizontal line represents the 95% CI for an individual effect size, and the yellow point indicates the corresponding Hedges’ g estimate. The red diamond indicates the overall pooled effect, and the black horizontal line indicates the 95% prediction interval (PI). In the right-hand subgroup panels, small points represent individual effect sizes, whereas larger points with black horizontal lines represent subgroup estimates and their 95% CIs. Values to the left of zero favour prolonged sitting, whereas values to the right of zero favour sitting interruptions

Memory function emerged as another significant positive outcome in this study, encompassing 10 studies and 53 effect sizes. A three-level meta-analysis showed that, compared with uninterrupted sitting, introducing brief standing or physical activity breaks during sedentary periods produced a pooled effect size for memory function of Hedges’ *g* = 0.37 (95% *CI*: 0.13 to 0.61, *p* = 0.003), indicating a small-to-moderate beneficial effect. Heterogeneity decomposition showed no within-study heterogeneity (*I*²_level 2 = 0%) but substantial between-study heterogeneity (*I*²_level 3 = 65.57%). Moreover, the prediction interval crossed the null (*PI* = − 0.51 to 1.25), suggesting that the effect may not be stable across study contexts. Given the low certainty of the evidence, a more cautious interpretation is that interruptions to prolonged sitting may have acute beneficial effects on memory function, although the consistency of this effect across studies remains uncertain Fig. 5.

**Fig. 6:**
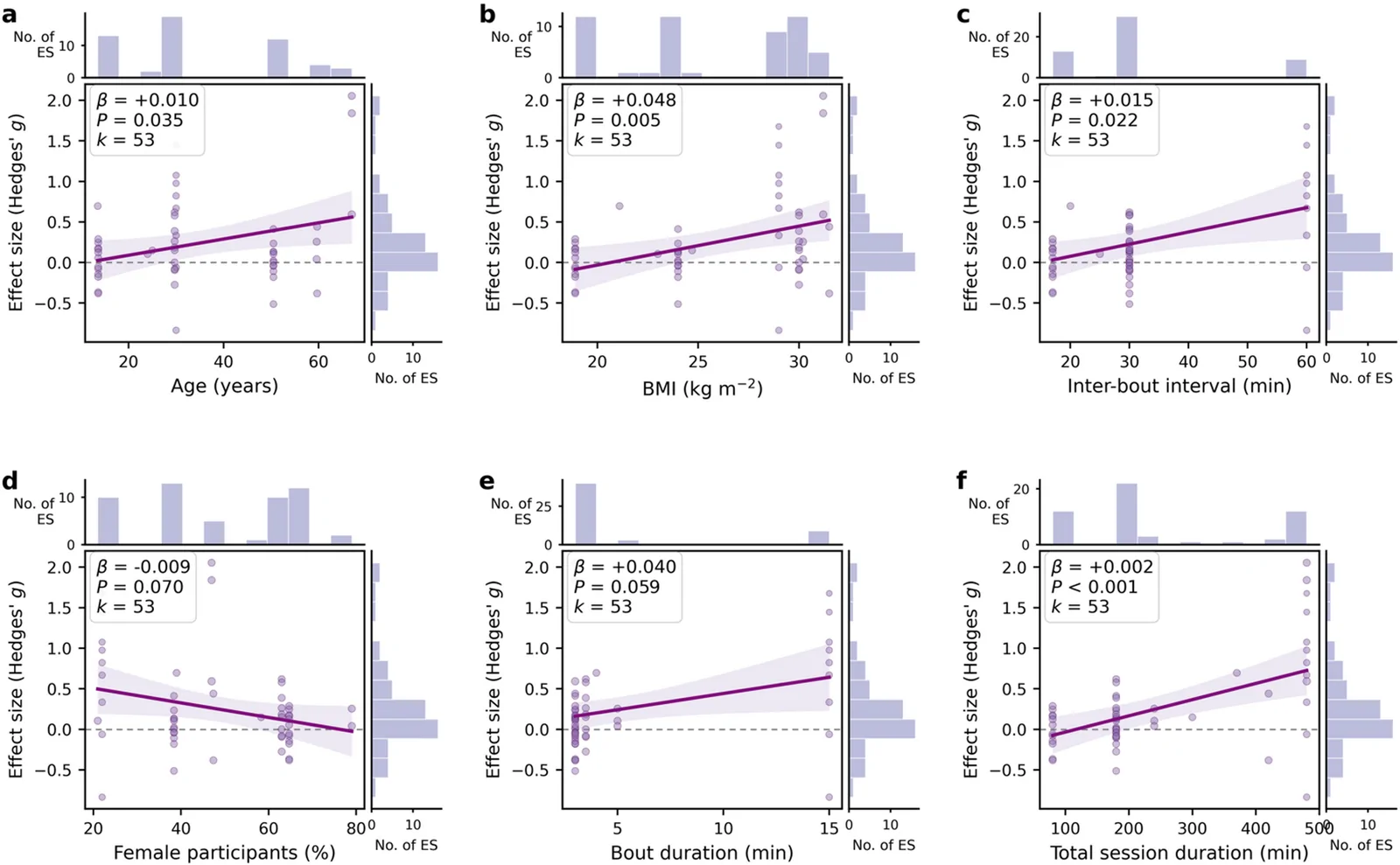
Meta-regression analyses of moderators of Memory Function.Panels represent: **a** age; **b** BMI; **c** inter-bout interval; **d** percentage of female participants; **e** bout duration; and **f** total experimental session duration. Each point represents one effect size. The solid purple line indicates the fitted meta-regression line, and the shaded band represents the 95% confidence interval. The horizontal dashed line marks the null effect (g = 0). Marginal histograms show the distributions of moderator values along the top axis and effect sizes along the right axis, expressed as the number of effect sizes (No. of ES). Each panel reports the regression coefficient (β), p value, and number of effect sizes (k)

Exploratory subgroup analyses suggested that age group (*p* < 0.001), BMI (*p* = 0.004), interruption type (*p* = 0.041), and exercise intensity (*p* = 0.004) may be associated with the magnitude of the effect size. Descriptive findings indicated more consistent improvements among young adults. Although the subgroup of older adults showed a larger point estimate (*g* = 1.47), this estimate was derived primarily from a single study and should therefore be interpreted with particular caution. Participants with overweight or obesity, walking- or standing-based interruptions, and low-intensity activities yielded relatively larger positive point estimates. Exploratory meta-regression further showed that age (*β* = 0.010, *p* = 0.035), BMI (*β* = 0.048, *p* = 0.005), interruption frequency (*β* = 0.015, *p* = 0.022), and experimental exposure duration (*β* = 0.002, *p* < 0.001) were positively associated with memory-related effect sizes, whereas no clear associations were observed for single-interruption duration or gender ratio. Given the substantial heterogeneity across studies and the limited number of studies in certain subgroups, these findings should be interpreted as preliminary indications of potential moderators and as hypotheses for future validation, rather than as robust evidence of dose-response relationships Fig. 6.

#### Information processing

The outcome of information processing was assessed in 4 studies comprising 18 effect sizes (Supplementary 4). A three-level meta-analysis showed that, compared with uninterrupted sitting, introducing brief standing or physical activity breaks during sedentary periods was associated with a pooled effect size of Hedges’ *g* = 0.32 (95% *CI*: −0.21 to 0.86, *p* = 0.221), indicating no clear evidence of improvement at present. Heterogeneity decomposition showed no within-study heterogeneity (*I*²_level 2 = 0%) but substantial between-study heterogeneity (*I*²_level 3 = 64.14%). In addition, the prediction interval was wide and crossed the null (*PI* = − 1.11 to 1.75), suggesting that the current estimate lacks stability. Given the low certainty of the evidence for this outcome, it is more appropriate to characterize the finding as indicating insufficient evidence, rather than clear evidence of no effect.

Although the overall pooled effect did not show a clear signal, we proceeded, in accordance with our pre-specified protocol, to conduct exploratory moderator analyses to examine potential sources of residual heterogeneity. In the exploratory subgroup analyses (Supplementary 6), only experimental setting showed a statistically significant effect (*p* = 0.037). Age group, BMI, interruption type, and exercise intensity showed no clear effects. Similarly, exploratory meta-regression analyses (Supplementary 7) did not identify any clear associations between effect sizes and age, BMI, interruption frequency, gender ratio, individual break duration, or total experimental duration. Given the limited number of studies examining this outcome and the uncertainty surrounding the overall effect, these exploratory findings should primarily be viewed as informing directions for future research.

#### Attention

The outcome of attention was assessed in 5 studies comprising 14 effect sizes (Supplementary 4). A three-level meta-analysis showed that, compared with uninterrupted sitting, introducing brief standing or physical activity breaks during sitting yielded a pooled effect size for attention of Hedges’ *g* = − 0.08 (95% *CI*: −0.38 to 0.22, *p* = 0.575), indicating no clear evidence of an acute improvement. Heterogeneity decomposition showed no within-study heterogeneity (*I*²_level 2 = 0%) and low-to-moderate between-study heterogeneity (*I*²_level 3 = 25.91%). The prediction interval crossed the null (*PI* = − 0.63 to 0.46), further suggesting uncertainty regarding the effect. Given the low certainty of the evidence, the most appropriate conclusion at this stage is that the acute effects of sitting interruptions on attention remain unclear.

Exploratory subgroup analyses provided no evidence of consistent moderators (Supplementary 6): age group (*p* = 0.056), BMI (*p* = 0.070), interruption type (*p* = 0.951), and interruption intensity (*p* = 0.212) were all non-significant. Exploratory meta-regression analyses (Supplementary 7) showed that age (*β* = −0.011, *p* = 0.022), BMI (*β* = −0.058, *p* = 0.017), and experimental exposure duration (*β* = −0.002, *p* = 0.015) were negatively associated with effect size, whereas no clear associations were observed for interruption frequency, gender ratio, or individual interruption duration.

### Risk of publication bias and sensitivity analysis

Sensitivity analyses generally supported the robustness of the conclusions from the primary analysis (Supplementary 8). Specifically, the direction and statistical significance of the main pooled effects remained largely unchanged after varying the paired-difference correlation between *r* = 0.3 and *r* = 0.7 (used to reconstruct the variance of within-participant paired comparisons; Methods, paragraph (a)) and after varying the within-study dependency correlation between *r* = 0.4 and *r* = 0.8 (used to impute the off-diagonal elements of the sampling variance–covariance matrix; Methods, paragraph (b)). The conclusions also remained broadly consistent across leave-one-out analyses, with no individual study appearing to disproportionately influence the pooled effects. In addition, the individual exclusion of each WebPlotDigitizer-extracted study did not appreciably alter the direction or statistical significance of the pooled effects, suggesting that the findings were not unduly influenced by digital data-extraction methods.

Formal tests for funnel plot asymmetry or small-study effects were applied only to outcomes for which the number of independent studies met a prespecified threshold (≥ 10 independent studies or study clusters). Based on the distribution of evidence included in this study, executive function (16 studies) and memory function (10 studies) met this criterion. In contrast, global cognition, information processing, and attention included only 2, 4, and 5 studies, respectively. Accordingly, formal Egger’s regression tests were not conducted for these outcomes, and no statistical inferences regarding publication bias were drawn.

**Fig. 7:**
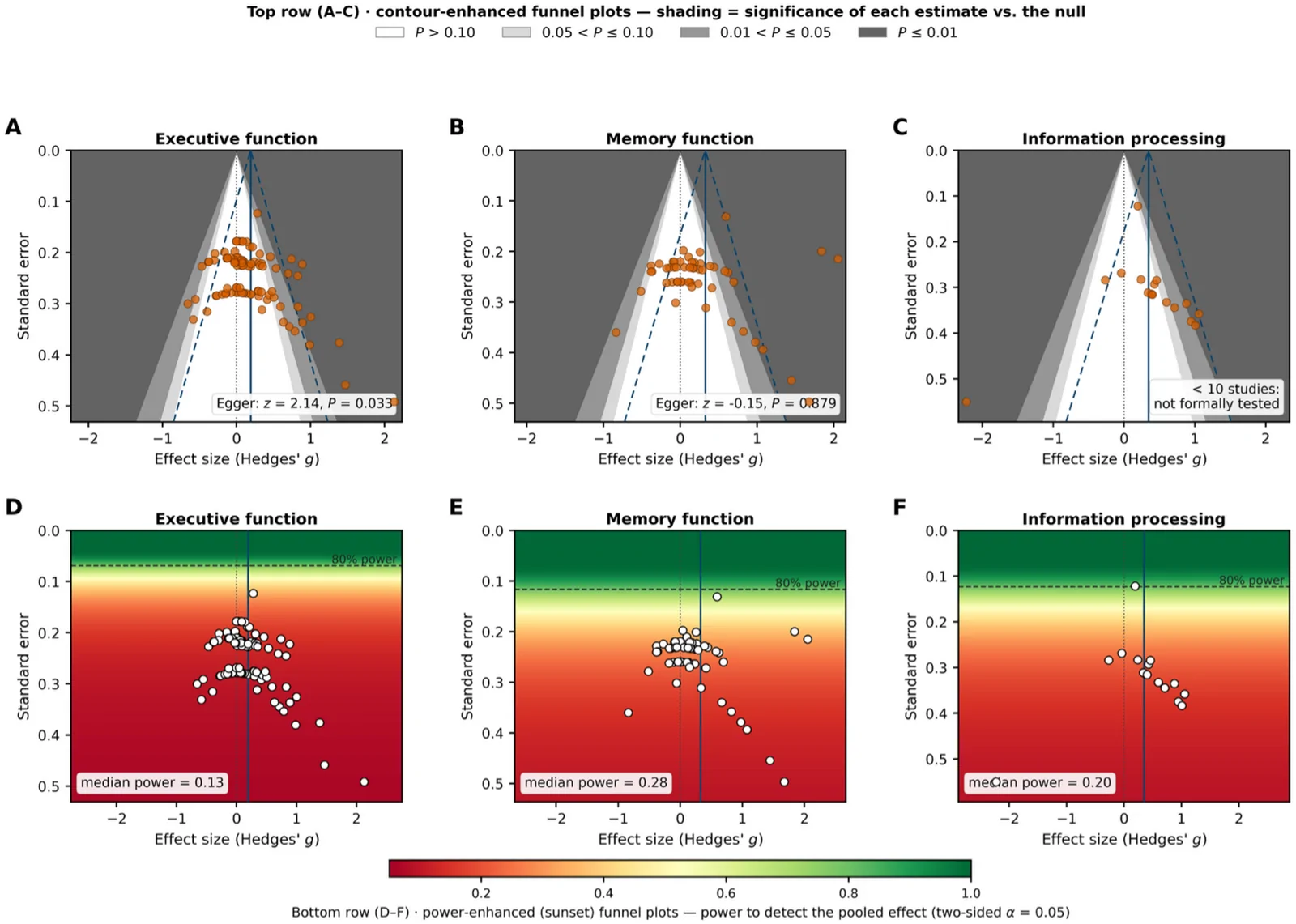
Top: contour-enhanced funnel plots; bottom: power-enhanced (sunset) funnel plots. **A**/**D**, Executive Function; **B**/**E**, Memory Function; **C**/**F**, Information Processing

Among the outcomes that met the criteria for formal testing, executive function showed evidence suggestive of funnel plot asymmetry or potential small-study effects (*z* = 2.14, *p* = 0.033). By contrast, memory function showed no clear evidence of asymmetry (*z* = − 0.15, *p* = 0.879;). Because this study included randomized crossover trials and retained multiple correlated effect sizes within individual studies, these results are best interpreted as suggestive indicators of small-study effects rather than as definitive evidence of publication bias. For global cognition, information processing, and attention, the number of independent studies was insufficient for formal testing; therefore, this paper provides only a descriptive evaluation based on visual inspection of the funnel plots, without drawing formal statistical inferences (Supplementary 9) (Fig. 7).

### GRADE analyses

The GRADE summary of findings (Supplementary 10) indicated that the overall certainty of evidence for the primary outcomes in this study was predominantly low. In particular, the outcome of global cognition was rated as very low certainty because of the sparse evidence base and unstable estimates. Unlike previous approaches that relied on fixed thresholds for downgrading, this study applied the standard GRADE framework to provide a holistic assessment of each outcome, taking into account risk of bias, inconsistency, indirectness, imprecision, and publication bias or small-study effects.

Specifically, the outcome of global cognition included only two randomized crossover trials, and the 95% confidence interval was wide and crossed the null, indicating highly unstable estimates. Accordingly, the evidence was downgraded for risk of bias and very serious imprecision, resulting in a final rating of very low certainty. Although executive function showed a statistically significant positive effect, the included studies were generally judged to have “some concerns” regarding risk of bias, and formal testing suggested potential small-study effects; therefore, the certainty of evidence for this outcome was rated as low. Memory function likewise showed a statistically significant improvement; however, substantial heterogeneity across studies, together with a prediction interval crossing the null, suggested limited stability of the effect across study contexts. Therefore, the certainty of evidence for this outcome was also rated as low. For both information processing and attention, the number of included studies was limited, and the 95% confidence intervals for the pooled effects crossed the null, indicating imprecise estimates. Consequently, the certainty of evidence for both outcomes was rated as low.

Overall, the current evidence suggests that interruptions to prolonged sitting may have acute beneficial effects on executive function and memory function. However, because the certainty of evidence for most outcomes remains low or very low, these findings should be interpreted cautiously and should not be used to support overly strong prescriptive recommendations.

## Discussion

**Fig. 8:**
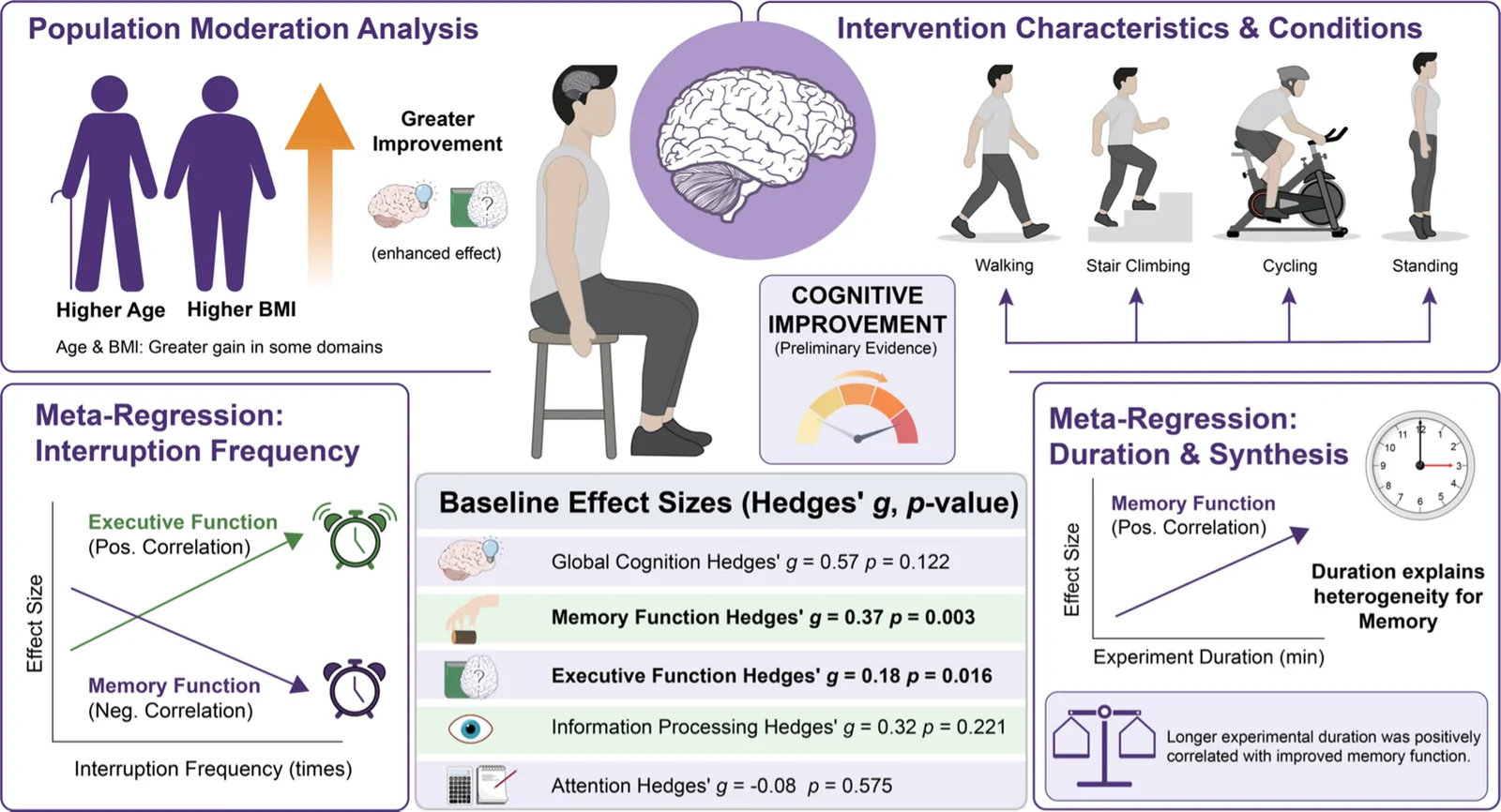
Evidence Summary. NOTE: Graphical summary of the main findings. The central panel shows pooled Hedges’ g and p-values across five cognitive domains; bold values indicate statistically significant effects. Exploratory meta-regression suggested that higher age and higher BMI were associated with greater cognitive gain in some domains, although associations varied across domains and were not uniformly positive (top-left). Meta-regression indicated that interruption frequency was positively associated with executive function but negatively with memory function (bottom-left), whereas longer experimental duration was positively associated with memory function (bottom-right). All moderator analyses are exploratory and hypothesis-generating

This systematic review and three-level meta-analysis showed evidence of acute cognitive effects primarily in two domains: executive function and memory function. Executive function showed a small positive effect, whereas memory function showed a small-to-moderate positive effect. In contrast, no consistent evidence of improvement was found for information processing or attention. Furthermore, global cognition outcomes, which were derived from a very limited number of studies and were characterized by wide confidence intervals and very low certainty of evidence, are best treated as supplementary findings rather than as central to the primary narrative of this study. Taken together, these findings do not indicate a broad cognitive benefit of sitting interruptions. Rather, they suggest that executive function and memory function may be the cognitive domains most likely to show acute responses, while the overall certainty of evidence remains low. The results should therefore be interpreted as hypothesis-supporting evidence for domain-specific acute effects, not as definitive evidence of intervention efficacy (Fig. 8).

### Main findings: domain-specific effects on cognitive function

The findings of this study indicate that interruptions to prolonged sitting are associated with acute improvements in executive function (Hedges’ g = 0.18, *p* = 0.016) and memory function (Hedges’ g = 0.37, *p* = 0.003), and the overall direction of these effects is consistent with that reported by Feter et al. [49]. These domain-specific effects may be partly explained by acute changes in cerebral hemodynamics, metabolic regulation, and neurotrophic signaling, although direct mechanistic evidence remains limited. First, neurovascular coupling and cerebral hemodynamics may play central roles. Prolonged uninterrupted sitting promotes venous pooling in the lower extremities, reduces venous return, and attenuates cardiac output, collectively impairing cerebral perfusion pressure. Empirical evidence indicates that 3–4 h of continuous sitting can reduce middle cerebral artery blood flow velocity by 5%–6% [27, 102], a hemodynamic deficit with documented implications for executive function and memory performance [102]. By contrast, sitting interruptions intermittently impose cardiovascular stress that generates pulsatile shear forces within the cerebral vasculature. These episodic hemodynamic perturbations are particularly consequential for the prefrontal cortex and hippocampus regions that are richly vascularized and acutely sensitive to fluctuations in cerebral blood flow, where pulsatile shear may potentiate neurovascular coupling and support activity-dependent neural plasticity [103]. Concurrently, transient elevations in heart rate and metabolic demand augment laminar shear stress at the endothelial surface, stimulating endothelial nitric oxide synthase (eNOS) activity and enhancing the bioavailability of the vasodilator nitric oxide (NO) [104, 105]. This shear-mediated upregulation of NO signaling sustains flow-mediated vasodilation, counteracts the endothelial dysfunction associated with prolonged inactivity [106, 107], and may thereby preserve the cerebrovascular reactivity necessary for optimal cognitive performance [104, 105].

Neurotrophic and metabolic pathways may provide additional, but still indirect, explanations. Studies by Heiland et al. suggest that even a single bout of acute exercise can increase peripheral BDNF levels [60], and interruptions to prolonged sitting may likewise provide a potent stimulus for acute or sustained elevations in BDNF. Because BDNF promotes neurogenesis, synaptic plasticity, and neuronal survival, it provides an important molecular basis for memory consolidation and cognitive enhancement [108, 109]. In addition, glucose is a critical substrate for cerebral energy metabolism, and dysregulated glucose levels have been associated with poorer cognitive performance [110, 111]. Research by Chandrasekaran et al. [112] suggests that interruptions to prolonged sitting can improve peripheral glucose regulation, which may indirectly support cognitive performance by optimizing cortical glucose utilization. Consistent with this interpretation, the SBAE global consensus led by Yin et al. [32] concluded that this strategy is supported by strong evidence for improving glycemic outcomes, providing a physiologically coherent explanation for its observed benefits for executive and memory functions. However, most included studies did not directly measure cerebral perfusion, BDNF, glucose dynamics, or inflammatory markers alongside cognitive outcomes. Therefore, these mechanisms should be viewed as biologically plausible explanations rather than confirmed mediating pathways.

In contrast, the present study did not identify significant improvements associated with interruptions to prolonged sitting in global cognition (Hedges’ *g* = 0.57, *p* = 0.122), information processing (Hedges’ *g* = 0.32, *p* = 0.221), or attention (Hedges’ *g* = − 0.08, *p* = 0.575). This pattern may reflect genuine domain-specific sensitivity, but it may also reflect limited statistical power, heterogeneous task paradigms, and differences in measurement sensitivity. Executive and memory functions rely more heavily on highly plastic fronto-hippocampal pathways and appear more sensitive to short-term fluctuations in cerebral blood flow and neurotrophic signaling, such as the BDNF/TrkB pathway [113, 114]. They may therefore be more likely to benefit from acute or short-cycle interruptions to prolonged sitting involving physical activity. By contrast, attention and information processing may depend more strongly on subcortical structures, white matter integrity, and basic sensorimotor processing speed [115]. Accordingly, the absence of statistically significant effects in these domains should not be interpreted as evidence that sitting interruptions are ineffective for attention or information processing; rather, the current evidence remains too sparse and heterogeneous to support a stable conclusion.

It should also be noted that, although the present study did not detect significant improvements in global cognition following interruptions to prolonged sitting, a finding consistent with that of Li et al. [50], this result should be interpreted cautiously. One possible explanation lies in differences in measurement tools. Previous studies have often used total scores from clinical screening scales, such as the MMSE or MoCA, to represent global cognition [116], whereas the present study [82, 85] used a composite index derived from a broader battery of cognitive tasks. These two types of indicators may differ in construct coverage and measurement sensitivity, thereby reducing comparability and increasing heterogeneity. Future research should adopt comparable measures of global cognition within a unified conceptual framework and use cross-validation across multiple instruments to improve the interpretability and comparability of findings.

Our acute findings complement the recent lifespan synthesis by Zou et al. [43], which concluded that habitual sedentary behaviour is associated with subtle, heterogeneous decrements in cognitive performance and brain structure across the lifespan and emphasised that the type of sedentary behaviour, particularly whether it is cognitively active or cognitively passive, may moderate these associations. Whereas that synthesis is built largely on chronic, observational evidence, the present meta-analysis shows that even acute, single-session interruptions to prolonged sitting can transiently shift performance in specific domains (executive and memory function). The two lines of evidence are therefore complementary: acute experimental data provide a proximal, mechanistically plausible counterpart to the chronic associations described across the lifespan, while the lifespan perspective underscores that the cognitive context of the sedentary behaviour being interrupted (active vs. passive) is itself likely to shape the response, an issue we return to in our discussion of future directions.

### Potential moderators of cognitive effects: explanatory frameworks and research directions

Feter et al. [49], in a meta-analysis covering diverse age groups, suggested that reducing sedentary behavior and increasing physical activity may benefit cognitive function. However, their research primarily examined the broad effects of general sedentary reduction on brain and cognitive outcomes, rather than the acute behavioral strategy of introducing interruptions to prolonged sitting. Moreover, it did not systematically evaluate the potential influence of interruption parameters, such as frequency, duration, modality, and experimental context, on cognitive outcomes. The present review therefore offers a more focused view of potential effect modifiers within randomized crossover trials of sitting interruptions. However, these moderator findings should be interpreted at the study level. Because participant characteristics, interruption modality, frequency, intensity, and setting were not independently randomized across studies, the observed patterns are best understood as explanations for heterogeneity and as leads for future experimental testing, rather than as evidence of causal effect modification or optimal protocols.

#### Participant characteristics

The exploratory analyses in this study suggest that age and BMI may be associated with the effect sizes observed for executive function and memory function. Overall, the magnitude of improvement appeared to be greater among older adults and individuals with overweight or obesity. One possible interpretation is that these groups may have greater physiological susceptibility to prolonged sitting and therefore more room for short-term improvement when sitting is interrupted. With advancing age, vascular endothelial function, neurovascular coupling, and neuroplasticity gradually decline [117]. Similarly, overweight and obesity are often accompanied by insulin resistance and chronic low-grade inflammation, both of which have been linked to early cognitive impairment [118]. Interruptions to prolonged sitting have been reported to improve acute metabolic responses and attenuate adverse physiological states associated with prolonged sitting, for example by enhancing insulin sensitivity and reducing inflammatory markers [119, 120].

Nevertheless, this interpretation remains indirect. Some subgroup estimates were based on a small number of studies, and the included trials differed in participant characteristics, cognitive tasks, and interruption protocols. Moreover, most studies did not measure cerebral blood flow, metabolic markers, or inflammatory indicators together with cognitive outcomes. Age and BMI should therefore be treated as potential effect modifiers for future investigation, not as a basis for population-specific recommendations at this stage.

#### Interruption protocol characteristics

This study further highlights the potential role of interruption protocol characteristics in explaining between-study heterogeneity. Feter et al. suggested that multiple sitting interruptions yield small but significant cognitive benefits, whereas a single interruption does not reach statistical significance (0.20 vs. 0.08) [49]. Extending this work, the present review examined whether specific features of sitting-interruption protocols, including frequency, modality, intensity, session duration, and setting, might help explain variation in cognitive effects. These analyses were exploratory and study-level in nature; therefore, they should not be interpreted as identifying optimal interruption prescriptions.

Among the protocol characteristics examined, interruption frequency showed one of the clearest exploratory associations with effect size. Specifically, shorter intervals between interruptions were associated with larger executive-function effects, whereas memory-function effects appeared larger in studies using longer intervals. This divergence may reflect differences in how executive and memory-related tasks respond to repeated arousal, hemodynamic stimulation, and task-context disruption, but this explanation remains speculative because frequency was confounded with other protocol and study characteristics across trials. From a neurobiological perspective, executive function depends substantially on frontoparietal control networks, whose performance may be sensitive to short-term fluctuations in cerebral perfusion and neuromodulatory state [121]. Carter et al. [27] provided indirect support for this mechanism using transcranial Doppler ultrasound: under conditions of equivalent total activity duration, 2 min of light walking every 30 min attenuated the decline in cerebral arterial blood flow velocity, whereas 8 min of walking every 2 h had no significant effect. By contrast, memory-related outcomes may be more sensitive to the timing of activity in relation to encoding, maintenance, and consolidation. A study by Dongen et al. [122] suggested that the memory-enhancing effects of exercise depend substantially on both dose and timing: exercise performed approximately 4 h after memory encoding improved retention over the subsequent 48 h, whereas exercise performed immediately after encoding did not produce comparable benefits. Frequent interruptions may also impose task-switching costs or contextual reconstruction demands, particularly for memory tasks involving encoding or maintenance processes [123]. Interestingly, interruption frequency has also shown potential relevance for other health outcomes. Yin et al. [124] reported that, compared with low-frequency interruption protocols, high-frequency protocols produced superior acute effects on several cardiometabolic markers. Over the longer term, Grgic et al. [125] likewise noted that higher training frequencies generally outperform lower frequencies. These findings point to a broader possibility: different cognitive outcomes may not respond to the same interruption pattern in the same way. However, the present evidence is not sufficient to define different protocols for different cognitive domains. Direct head-to-head randomized crossover trials that isolate frequency while holding total activity dose, intensity, task context, and assessment timing constant are needed before dose-response conclusions can be drawn.

In addition to interruption frequency, the form and intensity of the interruption may also contribute to between-study heterogeneity. Among the studies included in this review, walking was the most frequently studied modality and showed a relatively consistent positive signal for both executive function (g = 0.12) and memory function (g = 0.35). Low-intensity physical activity also showed a larger point estimate for memory function (g = 0.54) and a positive, although non-significant, estimate for executive function (g = 0.22, *p* = 0.07). These patterns are compatible with the idea that low-intensity movement may provide sufficient arousal and hemodynamic stimulation without imposing substantial fatigue, discomfort, or task disruption. Activities such as resistance exercise and cycling typically involve higher muscular or cardiovascular demands, during which blood flow may be redistributed to skeletal muscle and the heart to sustain oxygen delivery, potentially limiting cerebral perfusion in the prefrontal cortex [126]. From a feasibility perspective, intermittent walking is also more compatible with everyday settings than more demanding exercise modalities. A substantial body of research suggests that incorporating walking breaks into prolonged sitting can reduce cardiometabolic risk [127, 128], and some studies have also reported cognitive benefits. Notably, Hamilton et al. [129] proposed that soleus-dominant ankle plantarflexion, a seated micro-activity, may help stabilize blood glucose and thus offers a novel strategy for mitigating the adverse effects of sedentary behavior. In the present review, the relatively large effect size observed for jiggling suggests that low-load lower-limb micro-activities may merit further investigation. However, because this estimate was derived from a single study, it should be viewed as a signal for replication rather than evidence of efficacy.

Beyond the prescribed activity parameters themselves, study implementation characteristics may also shape observed cognitive effects. Longer experimental sessions were associated with larger memory-function effects, possibly because the adverse physiological consequences of prolonged sitting accumulate over time and because memory-related benefits of activity may depend on the timing of assessment relative to encoding or consolidation. The larger executive-function estimate observed in simulated real-world settings than in laboratory-based settings may also suggest that task relevance, motivation, and environmental context influence cognitive responsiveness. However, this contextual pattern was based on very limited evidence and may reflect study-specific features rather than a general setting effect. Overall, the observed patterns for frequency, intensity, modality, session duration, and setting should be treated as provisional explanations for heterogeneity. They are useful for designing future trials, but they do not establish dose-response relationships or standardized protocols for sitting interruptions.

### Clinical significance and practical applications

The clinical and practical significance of this study is best understood as identifying a feasible supplementary strategy, rather than establishing a prescriptive regimen. Based on the current evidence, incorporating brief standing or physical activity breaks during prolonged sitting may be associated with small acute improvements in executive function and memory function. For people engaged in office work, academic study, or other contexts characterized by high sedentary exposure, sitting interruptions may therefore represent a low-barrier option that can be integrated into daily routines without requiring structured exercise sessions or specialized facilities.

However, the current evidence remains insufficient to support definitive recommendations regarding optimal interruption frequency, single-bout duration, or preferred modality. Although walking, low-intensity activity, and some interruption-frequency patterns showed promising exploratory signals, these findings are constrained by the limited number of studies, subgroup imbalance, heterogeneity in cognitive tasks, and low certainty of evidence. Accordingly, simple strategies such as standing, brief walking, or low-load lower-limb movements may be considered feasible options for breaking up prolonged sitting, but their relative efficacy and optimal implementation parameters remain uncertain.

Furthermore, sitting interruptions should not be regarded as a substitute for structured physical activity. Their main practical value lies in providing intra-day microdoses of movement that may complement, rather than replace, guideline-recommended physical activity. This distinction is important for translation: sitting interruptions may be particularly useful for reducing prolonged sedentary exposure in real-world contexts, but the present evidence does not justify presenting them as a stand-alone cognitive enhancement intervention.

### Future research directions: measurement, context, and within-person dynamics

Future research should strengthen both behavioural measurement and cognitive assessment. Although controlled crossover trials can prescribe the timing, frequency, and duration of interruptions, the actual movement behaviour performed before, during, and after experimental sessions is often incompletely captured. Combining protocol-based experimental control with device-based measurement, such as thigh-worn inclinometers, accelerometers, heart-rate monitoring, or wearable physiological sensors, would allow researchers to verify sitting, standing, stepping, intensity, and compliance in real time. Given the small number of available studies and the heterogeneity in tasks, time points, and protocols, an individual participant data meta-analysis would also be valuable. Such an approach could harmonize raw cognitive outcomes, model repeated measurements more consistently, and test whether age, sex, BMI, baseline cognitive performance, cardiometabolic status, or habitual sedentary behaviour modifies acute responses to sitting interruptions.

Future studies should also account for the cognitive context of the sedentary behaviour being interrupted. The present review necessarily treated prolonged sitting as the common comparator, but sedentary behaviour is not cognitively homogeneous. Mentally passive sedentary behaviours, such as television viewing, may differ from mentally active sedentary behaviours, such as reading, computer-based work, or cognitively demanding study, in their relationships with mental health and cognitive outcomes [130]. This distinction is important because interrupting mentally passive sitting may primarily increase arousal and physiological activation, whereas interrupting mentally active sitting may also disrupt task continuity, memory encoding, or sustained attentional engagement. Real-world studies should therefore combine device-based movement measurement with repeated mobile or ambulatory cognitive assessments delivered through smartphones or tablets. Ambulatory cognitive assessment has been shown to provide reliable and valid repeated measures of cognitive performance in everyday contexts [131], and could help determine whether naturally occurring or prompted sitting interruptions are followed by short-term changes in executive function, memory-related performance, attention, or processing efficiency.

Finally, future designs should move beyond between-person comparisons and examine within-person dynamics. The present three-level meta-analysis addressed dependency among multiple effect sizes within the same study, but it could not determine whether a given individual performs better on occasions when their own prolonged sitting is interrupted than on occasions when it is not. Microrandomized trials, ecological momentary intervention designs, and within-person crossover designs embedded in daily life could address this question more directly by randomizing prompts after sedentary bouts of different durations and administering brief cognitive assessments before and after selected prompts. A recent within-person encouragement study provides a concrete example of this direction: Giurgiu et al. used brief sedentary-break prompts in daily life to examine causal effects on affective and cognitive parameters, illustrating how sedentary-break research can move toward ecologically valid within-person causal designs [132]. These designs would also allow time of day, sleep-wake state, prior food intake, fatigue, mood, circadian preference, and accumulated cognitive load to be measured and modelled. This is important because apparent intervention effects may otherwise partly reflect temporal variation in cognitive performance rather than the sitting-interruption protocol itself.

### Strengths and limitations of the study

Under relatively stringent inclusion criteria, this study focused on the acute effects of brief standing or physical activity breaks during prolonged sitting on cognitive function. In addition, by applying a three-level random-effects model combined with robust variance estimation to address effect-size dependency arising from multiple tasks and time points within the same study, the analysis improved the comprehensiveness of the evidence synthesis. The study also conducted stratified analyses across five cognitive domains and further performed exploratory moderator analyses to identify potential sources of heterogeneity. However, these strengths do not indicate that the current evidence is sufficient to support strong prescriptive conclusions, and the following limitations warrant careful consideration.

First, the overall sample size of the included studies remains limited, and the number of independent studies varies substantially across outcomes. In particular, the evidence base for global cognition, information processing, and attention remains relatively weak, resulting in less stable pooled estimates, wider prediction intervals, and lower certainty of evidence. Second, although the three-level model allowed multiple effect sizes within the same study to be retained, this approach also means that individual studies, particularly those reporting many tasks or measurement time points, may exert disproportionate influence on the overall results. Although we sought to reduce this risk through robust variance estimation and sensitivity analyses, the potential impact of the specified within-study dependency structure on the precision of the results cannot be fully excluded. Third, effect-size construction for crossover trials depends, to some extent, on the specification of correlation coefficients and the completeness of reported statistics. For studies that did not directly report paired-comparison data, values had to be back-calculated, or correlation parameters imputed from the available information. Therefore, although robustness was examined through sensitivity analyses, these assumptions may still introduce additional uncertainty into some outcomes. Fourth, although the classification of cognitive outcomes into specific domains was based on a pre-established task-to-domain coding scheme, cognitive tasks are not theoretically mutually exclusive. Individual tasks or subtasks often involve multiple cognitive components simultaneously. Accordingly, assigning complex tasks to a single cognitive domain necessarily involves simplification, which may blur the boundaries between findings across domains. Fifth, subgroup analyses and meta-regressions are primarily intended to generate research hypotheses rather than confirm optimal parameters. Some subgroups were supported by very few studies, or even a single study, resulting in limited statistical power and unstable estimates. Consequently, findings related to age, BMI, interruption modality, frequency, and experimental setting should be interpreted as potential clues rather than established patterns of effect modification. Sixth, the ability to assess publication bias and small-study effects was limited. Formal Egger’s tests could be applied only to a small number of outcomes for which the number of independent studies met the required threshold; for the remaining outcomes, robust formal assessment was not feasible. Therefore, the influence of small-study effects or selective reporting cannot be ruled out. Seventh, the available evidence is derived almost exclusively from acute randomized crossover trials, with most studies conducted in laboratory or simulated settings. Eighth, although intensity was coded at the experimental-condition level using ACSM criteria, protocols that combined breaks of different intensities within a single condition may still have introduced some classification uncertainty. In addition, although a washout period was required for inclusion and trials without a washout were excluded, incomplete washout cannot be fully ruled out in crossover designs, particularly when a higher-intensity activity condition preceded a lower-intensity or standing condition. Ninth, most included studies provided limited information on the cognitive or behavioural nature of the sedentary period being interrupted. As a result, we could not determine whether the effects of sitting interruptions differ between mentally passive sedentary contexts and mentally active contexts, such as computer work, reading, or study. Tenth, the included studies varied in the timing of cognitive assessments and did not always provide sufficient information on time of day, pre-test behaviours, or the delay between the final interruption and cognitive testing. These factors may influence cognitive performance and therefore add uncertainty to the interpretation of acute intervention effects. Finally, because the available evidence was based on aggregate study-level data, the present meta-analysis could not directly examine day-to-day within-person variability or individual-level responsiveness to sitting interruptions. As a result, the present study is better suited to addressing whether interruptions to prolonged sitting elicit acute cognitive responses than whether these effects translate into long-term, sustainable, and functionally meaningful cognitive benefits in real-world settings. Adherence, feasibility, behavioral compensation, and long-term outcomes in everyday contexts remain to be established.

These limitations indicate that the present findings should be used primarily to guide the design of more precise trials, rather than to support immediate protocol standardization. In particular, future work should clarify whether domain-specific cognitive responses depend on the type of sedentary behaviour interrupted, the timing of cognitive assessment, and within-person variability in daily movement behaviour.

## Conclusions

This systematic review and three-level meta-analysis of randomized crossover trials suggests that brief standing or physical activity breaks during prolonged sitting may be associated with small acute improvements in executive function and memory function. However, the certainty of evidence for these statistically significant findings was low, and the available evidence for global cognition, information processing, and attention remains insufficient and inconsistent, with very low certainty for global cognition. Therefore, the present findings should be interpreted as suggestive rather than definitive evidence of acute cognitive benefits. The observed differences across older adults, individuals with overweight or obesity, and varying interruption parameters should be regarded as exploratory and hypothesis-generating indicators of possible effect modification, rather than as a basis for precise prescriptive recommendations. Accordingly, at this stage, these interruptions to prolonged sitting may be considered a feasible complementary strategy with potential cognitive benefits, but the current evidence is not sufficient to establish a standardized protocol or optimal sitting-interruption prescription. Future studies with larger sample sizes, more rigorous designs, and more standardized outcome assessments are needed to confirm these findings and refine protocols for interrupting prolonged sitting. 

## Supplementary Information

Supplementary Material 1.

## Data Availability

No datasets were generated or analysed during the current study.

## Abbreviations

ACSM: American College of Sports Medicine
BDNF: Brain-derived neurotrophic factor
BMI: Body mass index
CI: Confidence interval
CR2: Bias-reduced small-sample correction for cluster-robust variance estimation
CRVE: Cluster-robust variance estimation
GRADE: Grading of Recommendations Assessment, Development, and Evaluation
FITT: Frequency, Intensity, Time, and Type (framework for characterizing physical activity)
HRmax: Maximum heart rate
HRR: Heart rate reserve
MET: Metabolic equivalent of task
ML: Maximum likelihood
MMSE: Mini-Mental State Examination
MoCA: Montreal Cognitive Assessment
MVC: Maximal voluntary contraction
PI: Prediction interval
PICOS: Population, Intervention, Comparison, Outcome, and Study Design
PRISMA: Preferred Reporting Items for Systematic Reviews and Meta-Analyses
PROSPERO: International Prospective Register of Systematic Reviews
REML: Restricted maximum likelihood
RoB 2: Risk of Bias 2 (revised Cochrane risk-of-bias tool for randomized trials)
RPE: Rating of perceived exertion
RVE: Robust variance estimation
SD: Standard deviation
SE: Standard error
SMD: Standardized mean difference
TMT: Trail Making Test (TMT-B: Trail Making Test, Part B)
