# Supplementary material for: Acute cognitive effects of interruptions to prolonged sitting with brief standing or physical activity breaks: a systematic review and three-level meta-analysis
Source: Int J Behav Nutr Phys Act. 2026 Jul 13;23:79. doi: 10.1186/s12966-026-01953-6 (PMC13520540; doi:10.1186/s12966-026-01953-6)
Supplement: Supplementary file 1 [file 12966_2026_1953_MOESM1_ESM.docx]

**Journal:** *International Journal of Behavioral Nutrition and Physical Activity*

**Title:** Acute Cognitive Effects of Interruptions to Prolonged Sitting with Brief Standing or Physical Activity Breaks: A Systematic Review and Three-Level Meta-Analysis

**Authors:** Mingnan Zhuang^1,2†^, Mingyue Yin^3†^, Zongnan Liu^4†^, Ningkun Xiao^5,6†^, Qing Yi^7^, Zhiwei Fan^8^, Henghao Yan^9^ , Xiupeng Li^10,^ Bitai Wu^11^, Shuhan Qiu^12^, Anqi Chen^13^, Chao Xie^1^, Chenghao Liu^1^, Xintong Zou^14*^, Donglin Shi^2*^

**Affiliations:** ^1^ Sports Training College, Tianjin University of Sport, Tianjin, China; ^2^ School of Sport and Human Body Science, Hebei Sport University, Shijiazhuang, China; ^3^ Exercise & Nutrition Research Program, The Mary MacKillop Institute for Health Research, Australian Catholic University, Melbourne, VIC, Australia;^4^ The Faculty of Education, The National University of Malaysia, Lingkungan Ilmu, Bangi, Selangor, Malaysia; ^5^ Department of Immunochemistry, Institution of Chemical Engineering, Ural Federal University, Yekaterinburg, Russia; ^6^ Laboratory for Brain and Neurocognitive Development, Department of Psychology, Institution of Humanities, Ural Federal University, Yekaterinburg, Russia; ^7^ Division of Sports Science and Physical Education, Tsinghua University, Beijing, China; ^8^ Faculty of Health Sciences and Sports, Macao Polytechnic University, Macao S.A.R., China; ^9^ Institute of Sport Science, Southwest University, Chongqing, China; ^10^ Department of Physical Education, China University of Mining and Technology (Beijing), Beijing, China; ^11^ School of Physical Education and Sports Science, Hengyang Normal University, Hengyang, Hunan, China; ^12^ East China Normal University, Shanghai, China; ^13^ Shandong Sport University, Jinan, China; ^14^ Department of Anesthesiology, The First Affiliated Hospital of Wenzhou Medical University, Wenzhou, China

**Supplementary Materials1. The PRISMA checklist**

**Supplementary Table 1.** PRISMA 2020 for Abstracts Checklist

| **Section and Topic** | **Item #** | **Checklist item** | **Reported (Yes/No)** |
| --- | --- | --- | --- |
| **TITLE** | | |  |
| Title | 1 | Identify the report as a systematic review. | Yes |
| **BACKGROUND** | | |  |
| Objectives | 2 | Provide an explicit statement of the main objective(s) or question(s) the review addresses. | Yes |
| **METHODS** | | |  |
| Eligibility criteria | 3 | Specify the inclusion and exclusion criteria for the review. | Yes |
| Information sources | 4 | Specify the information sources (e.g. databases, registers) used to identify studies and the date when each was last searched. | Yes |
| Risk of bias | 5 | Specify the methods used to assess risk of bias in the included studies. | Yes |
| Synthesis of results | 6 | Specify the methods used to present and synthesise results. | Yes |
| **RESULTS** | | |  |
| Included studies | 7 | Give the total number of included studies and participants and summarise relevant characteristics of studies. | Yes |
| Synthesis of results | 8 | Present results for main outcomes, preferably indicating the number of included studies and participants for each. If meta-analysis was done, report the summary estimate and confidence/credible interval. If comparing groups, indicate the direction of the effect (i.e. which group is favoured). | Yes |
| **DISCUSSION** | | |  |
| Limitations of evidence | 9 | Provide a brief summary of the limitations of the evidence included in the review (e.g. study risk of bias, inconsistency and imprecision). | Yes |
| Interpretation | 10 | Provide a general interpretation of the results and important implications. | Yes |
| **OTHER** | | |  |
| Funding | 11 | Specify the primary source of funding for the review. | No |
| Registration | 12 | Provide the register name and registration number. | Yes |

**Supplementary Table 2**. PRISMA 2020 Checklist

| **Section and Topic** | **Item #** | **Checklist item** | **Location where item is reported** |
| --- | --- | --- | --- |
| **TITLE** | | |  |
| Title | 1 | Identify the report as a systematic review. | 1 |
| **ABSTRACT** | | |  |
| Abstract | 2 | See the PRISMA 2020 for Abstracts checklist. | 2 |
| **INTRODUCTION** | | |  |
| Rationale | 3 | Describe the rationale for the review in the context of existing knowledge. | 3-4 |
| Objectives | 4 | Provide an explicit statement of the objective(s) or question(s) the review addresses. | 4-5 |
| **METHODS** | | |  |
| Eligibility criteria | 5 | Specify the inclusion and exclusion criteria for the review and how studies were grouped for the syntheses. | 6 |
| Information sources | 6 | Specify all databases, registers, websites, organisations, reference lists and other sources searched or consulted to identify studies. Specify the date when each source was last searched or consulted. | 5 |
| Search strategy | 7 | Present the full search strategies for all databases, registers and websites, including any filters and limits used. | 5 |
| Selection process | 8 | Specify the methods used to decide whether a study met the inclusion criteria of the review, including how many reviewers screened each record and each report retrieved, whether they worked independently, and if applicable, details of automation tools used in the process. | 6 |
| Data collection process | 9 | Specify the methods used to collect data from reports, including how many reviewers collected data from each report, whether they worked independently, any processes for obtaining or confirming data from study investigators, and if applicable, details of automation tools used in the process. | 6-7 |
| Data items | 10a | List and define all outcomes for which data were sought. Specify whether all results that were compatible with each outcome domain in each study were sought (e.g. for all measures, time points, analyses), and if not, the methods used to decide which results to collect. | 6-7 |
|  | 10b | List and define all other variables for which data were sought (e.g. participant and intervention characteristics, funding sources). Describe any assumptions made about any missing or unclear information. | 6-8 |
| Study risk of bias assessment | 11 | Specify the methods used to assess risk of bias in the included studies, including details of the tool(s) used, how many reviewers assessed each study and whether they worked independently, and if applicable, details of automation tools used in the process. | 8 |
| Effect measures | 12 | Specify for each outcome the effect measure(s) (e.g. risk ratio, mean difference) used in the synthesis or presentation of results. | 8 |
| Synthesis methods | 13a | Describe the processes used to decide which studies were eligible for each synthesis (e.g. tabulating the study intervention characteristics and comparing against the planned groups for each synthesis (item #5)). | 8-9 |
|  | 13b | Describe any methods required to prepare the data for presentation or synthesis, such as handling of missing summary statistics, or data conversions. | 7-8 |
|  | 13c | Describe any methods used to tabulate or visually display results of individual studies and syntheses. | 9-10 |
|  | 13d | Describe any methods used to synthesize results and provide a rationale for the choice(s). If meta-analysis was performed, describe the model(s), method(s) to identify the presence and extent of statistical heterogeneity, and software package(s) used. | 8-10 |
|  | 13e | Describe any methods used to explore possible causes of heterogeneity among study results (e.g. subgroup analysis, meta-regression). | 10 |
|  | 13f | Describe any sensitivity analyses conducted to assess robustness of the synthesized results. | 11 |
| Reporting bias assessment | 14 | Describe any methods used to assess risk of bias due to missing results in a synthesis (arising from reporting biases). | 11 |
| Certainty assessment | 15 | Describe any methods used to assess certainty (or confidence) in the body of evidence for an outcome. | 11 |
| **RESULTS** | | |  |
| Study selection | 16a | Describe the results of the search and selection process, from the number of records identified in the search to the number of studies included in the review, ideally using a flow diagram. | 12 |
|  | 16b | Cite studies that might appear to meet the inclusion criteria, but which were excluded, and explain why they were excluded. | 12 |
| Study characteristics | 17 | Cite each included study and present its characteristics. | 12 |
| Risk of bias in studies | 18 | Present assessments of risk of bias for each included study. | 12 |
| Results of individual studies | 19 | For all outcomes, present, for each study: (a) summary statistics for each group (where appropriate) and (b) an effect estimate and its precision (e.g. confidence/credible interval), ideally using structured tables or plots. | 13-15 |
| Results of syntheses | 20a | For each synthesis, briefly summarise the characteristics and risk of bias among contributing studies. | 13-15 |
|  | 20b | Present results of all statistical syntheses conducted. If meta-analysis was done, present for each the summary estimate and its precision (e.g. confidence/credible interval) and measures of statistical heterogeneity. If comparing groups, describe the direction of the effect. | 13-15 |
|  | 20c | Present results of all investigations of possible causes of heterogeneity among study results. | 13-15 |
|  | 20d | Present results of all sensitivity analyses conducted to assess the robustness of the synthesized results. | 15 |
| Reporting biases | 21 | Present assessments of risk of bias due to missing results (arising from reporting biases) for each synthesis assessed. | 15 |
| Certainty of evidence | 22 | Present assessments of certainty (or confidence) in the body of evidence for each outcome assessed. | 15 |
| **DISCUSSION** | | |  |
| Discussion | 23a | Provide a general interpretation of the results in the context of other evidence. | 16-20 |
|  | 23b | Discuss any limitations of the evidence included in the review. | 20-22 |
|  | 23c | Discuss any limitations of the review processes used. | 20-22 |
|  | 23d | Discuss implications of the results for practice, policy, and future research. | 19-22 |
| **OTHER INFORMATION** | | |  |
| Registration and protocol | 24a | Provide registration information for the review, including register name and registration number, or state that the review was not registered. | 5 |
|  | 24b | Indicate where the review protocol can be accessed, or state that a protocol was not prepared. | 5 |
|  | 24c | Describe and explain any amendments to information provided at registration or in the protocol. | 5 |
| Support | 25 | Describe sources of financial or non-financial support for the review, and the role of the funders or sponsors in the review. | 23 |
| Competing interests | 26 | Declare any competing interests of review authors. | 23 |
| Availability of data, code and other materials | 27 | Report which of the following are publicly available and where they can be found: template data collection forms; data extracted from included studies; data used for all analyses; analytic code; any other materials used in the review. | 23 |

**Supplementary Materials 2. Search strategy**

**(1) The search strategy in PubMed**

| **Step** | **Keywords** |
| --- | --- |
| #1 | Search:"break*"[Title/Abstract] OR "interrupt*"[Title/Abstract] OR "interrupted sitting"[Title/Abstract] OR "breaks in sitting"[Title/Abstract] OR "breaks from sitting"[Title/Abstract] OR "sit less"[Title/Abstract] OR "movement break"[Title/Abstract] OR "movement breaks"[Title/Abstract] OR "activity break"[Title/Abstract] OR "activity breaks"[Title/Abstract] OR "active break"[Title/Abstract] OR "active breaks"[Title/Abstract] OR "physical activity break"[Title/Abstract] OR "physical activity breaks"[Title/Abstract] OR "exercise break"[Title/Abstract] OR "exercise breaks"[Title/Abstract] OR "microbreak*"[Title/Abstract] OR "exercise snack"[Title/Abstract] OR "exercise snacks"[Title/Abstract] OR "activity snacks"[Title/Abstract] OR "walking break"[Title/Abstract] OR "walking breaks"[Title/Abstract] OR "light-intensity activity"[Title/Abstract] OR "light-intensity activity"[Title/Abstract] OR "accumulated exercise"[Title/Abstract] OR "interrupting prolonged sitting"[Title/Abstract] OR "short bouts of accumulated exercise"[Title/Abstract] OR "SBAE"[Title/Abstract] OR "breaks"[Title/Abstract] OR "exercise snacks"[Title/Abstract] |
| #2 | Search: "Sedentary Behavior"[MeSH Terms] OR "Sitting Position"[MeSH Terms] OR "sedentary"[Title/Abstract] OR "Sedentary Behavior"[Title/Abstract] OR "sedentary behaviors"[Title/Abstract] OR "sedentary behaviour"[Title/Abstract] OR "sedentary behaviours"[Title/Abstract] OR "sedentary time"[Title/Abstract] OR "prolonged sedentary time"[Title/Abstract] OR "sedentariness"[Title/Abstract] OR "sitting"[Title/Abstract] OR "sitting time"[Title/Abstract] OR "time spent sitting"[Title/Abstract] OR "time sitting"[Title/Abstract] OR "seated time"[Title/Abstract] OR "prolonged sitting"[Title/Abstract] OR "uninterrupted sitting"[Title/Abstract] OR "continuous sitting"[Title/Abstract] OR "seated rest"[Title/Abstract] |
| #3 | "Cognition"[MeSH Terms] OR "Executive Function"[MeSH Terms] OR "Attention"[MeSH Terms] OR "Memory"[MeSH Terms] OR "Learning"[MeSH Terms] OR "Intelligence"[MeSH Terms] OR "Reaction Time"[MeSH Terms] OR "Psychomotor Performance"[MeSH Terms] OR "cognit*"[Title/Abstract] OR "cognitive performance"[Title/Abstract] OR "cognitive function"[Title/Abstract] OR "cognitive functions"[Title/Abstract] OR "cognitive functioning"[Title/Abstract] OR "neurocognit*"[Title/Abstract] OR "neuro-cognitive"[Title/Abstract] OR "Executive Function"[Title/Abstract] OR "executive functions"[Title/Abstract] OR "executive functioning"[Title/Abstract] OR "executive control"[Title/Abstract] OR "cognitive control"[Title/Abstract] OR "Attention"[Title/Abstract] OR "attentional"[Title/Abstract] OR "attentiveness"[Title/Abstract] OR "attentional control"[Title/Abstract] OR "attention control"[Title/Abstract] OR "working memory"[Title/Abstract] OR "updating"[Title/Abstract] OR "updating memory"[Title/Abstract] OR "task switching"[Title/Abstract] OR "switch task"[Title/Abstract] OR "set shifting"[Title/Abstract] OR "cognitive flexibility"[Title/Abstract] OR "processing speed"[Title/Abstract] OR "information processing"[Title/Abstract] OR "Reaction Time"[Title/Abstract] OR "reaction times"[Title/Abstract] OR "response time"[Title/Abstract] OR "response times"[Title/Abstract] OR "psychomotor vigilance"[Title/Abstract] OR "PVT"[Title/Abstract] OR "Stroop"[Title/Abstract] OR "Flanker"[Title/Abstract] OR "go/no-go"[Title/Abstract] OR "go/no-go"[Title/Abstract] OR "n-back"[Title/Abstract] OR "nback"[Title/Abstract] OR "digit span"[Title/Abstract] OR "trail making"[Title/Abstract] OR "digit symbol substitution"[Title/Abstract] OR "DSST"[Title/Abstract] OR "symbol digit"[Title/Abstract] OR "SDMT"[Title/Abstract] OR "choice reaction time"[Title/Abstract] OR "simple reaction time"[Title/Abstract] |
| #4 **Combine** | #1 AND #2 AND #3 |

**(2) The search strategy in Web of Science Core Collection**

| **Step** | **Keywords** |
| --- | --- |
| #1 | Search: TS=("break*" OR "interrupt*" OR "interrupted sitting" OR "breaks in sitting" OR "breaks from sitting" OR "sit less" OR "movement break" OR "movement breaks" OR "activity break" OR "activity breaks" OR "active break" OR "active breaks" OR "physical activity break" OR "physical activity breaks" OR "exercise break" OR "exercise breaks" OR "microbreak*" OR "exercise snack" OR "exercise snacks" OR "activity snacks" OR "walking break" OR "walking breaks" OR "light-intensity activity" OR "light-intensity activity" OR "accumulated exercise" OR "interrupting prolonged sitting" OR "short bouts of accumulated exercise" OR "SBAE" OR "breaks" OR "exercise snacks") |
| #2 | Search: TS=("Sedentary Behavior" OR "Sitting Position" OR "sedentary" OR "Sedentary Behavior" OR "sedentary behaviors" OR "sedentary behaviour" OR "sedentary behaviours" OR "sedentary time" OR "prolonged sedentary time" OR "sedentariness" OR "sitting" OR "sitting time" OR "time spent sitting" OR "time sitting" OR "seated time" OR "prolonged sitting" OR "uninterrupted sitting" OR "continuous sitting" OR "seated rest") |
| #3 | Search: TS=("Cognition" OR "Executive Function" OR "Attention" OR "Memory" OR "Learning" OR "Intelligence" OR "Reaction Time" OR "Psychomotor Performance" OR "cognit*" OR "cognitive performance" OR "cognitive function" OR "cognitive functions" OR "cognitive functioning" OR "neurocognit*" OR "neuro-cognitive" OR "Executive Function" OR "executive functions" OR "executive functioning" OR "executive control" OR "cognitive control" OR "Attention" OR "attentional" OR "attentiveness" OR "attentional control" OR "attention control" OR "working memory" OR "updating" OR "updating memory" OR "task switching" OR "switch task" OR "set shifting" OR "cognitive flexibility" OR "processing speed" OR "information processing" OR "Reaction Time" OR "reaction times" OR "response time" OR "response times" OR "psychomotor vigilance" OR "PVT" OR "Stroop" OR "Flanker" OR "go/no-go" OR "go/no-go" OR "n-back" OR "nback" OR "digit span" OR "trail making" OR "digit symbol substitution" OR "DSST" OR "symbol digit" OR "SDMT" OR "choice reaction time" OR "simple reaction time") |
| #4 **Combine** | #1 AND #2 AND #3 |

**(3) The search strategy in Scopus**

| **Step** | **Keywords** |
| --- | --- |
| #1 | Search: TITLE-ABS-KEY(break* OR interrupt* OR "interrupted sitting" OR "breaks in sitting" OR "breaks from sitting" OR "sit less" OR "movement break" OR "movement breaks" OR "activity break" OR "activity breaks" OR "active break" OR "active breaks" OR "physical activity break" OR "physical activity breaks" OR "exercise break" OR "exercise breaks" OR microbreak* OR "exercise snack" OR "exercise snacks" OR "activity snacks" OR "walking break" OR "walking breaks" OR "light-intensity activity" OR "light-intensity activity" OR "accumulated exercise" OR "interrupting prolonged sitting" OR "short bouts of accumulated exercise" OR SBAE OR breaks OR "exercise snacks") |
| #2 | Search: TITLE-ABS-KEY("Sedentary Behavior" OR "Sitting Position" OR sedentary OR "Sedentary Behavior" OR "sedentary behaviors" OR "sedentary behaviour" OR "sedentary behaviours" OR "sedentary time" OR "prolonged sedentary time" OR sedentariness OR sitting OR "sitting time" OR "time spent sitting" OR "time sitting" OR "seated time" OR "prolonged sitting" OR "uninterrupted sitting" OR "continuous sitting" OR "seated rest") |
| #3 | Search: TITLE-ABS-KEY("Cognition" OR "Executive Function" OR "Attention" OR "Memory" OR "Learning" OR "Intelligence" OR "Reaction Time" OR "Psychomotor Performance" OR cognit* OR "cognitive performance" OR "cognitive function" OR "cognitive functions" OR "cognitive functioning" OR neurocognit* OR "neuro-cognitive" OR "Executive Function" OR "executive functions" OR "executive functioning" OR "executive control" OR "cognitive control" OR "Attention" OR attentional OR attentiveness OR "attentional control" OR "attention control" OR "working memory" OR updating OR "updating memory" OR "task switching" OR "switch task" OR "set shifting" OR "cognitive flexibility" OR "processing speed" OR "information processing" OR "Reaction Time" OR "reaction times" OR "response time" OR "response times" OR "psychomotor vigilance" OR PVT OR Stroop OR Flanker OR "go/no-go" OR "go/no-go" OR "n-back" OR nback OR "digit span" OR "trail making" OR "digit symbol substitution" OR DSST OR "symbol digit" OR SDMT OR "choice reaction time" OR "simple reaction time") |
| #4 **Combine** | #1 AND #2 AND #3 |

**(4) The search strategy in The Cochrane Library**

| **Step** | **Keywords** |
| --- | --- |
| #1 | Search: (break*):ti,ab OR (interrupt*):ti,ab OR ("interrupted sitting"):ti,ab OR ("breaks in sitting"):ti,ab OR ("breaks from sitting"):ti,ab OR ("sit less"):ti,ab OR ("movement break"):ti,ab OR ("movement breaks"):ti,ab OR ("activity break"):ti,ab OR ("activity breaks"):ti,ab OR ("active break"):ti,ab OR ("active breaks"):ti,ab OR ("physical activity break"):ti,ab OR ("physical activity breaks"):ti,ab OR ("exercise break"):ti,ab OR ("exercise breaks"):ti,ab OR (microbreak*):ti,ab OR ("exercise snack"):ti,ab OR ("exercise snacks"):ti,ab OR ("activity snacks"):ti,ab OR ("walking break"):ti,ab OR ("walking breaks"):ti,ab OR ("light-intensity activity"):ti,ab OR ("light-intensity activity"):ti,ab OR ("accumulated exercise"):ti,ab OR ("interrupting prolonged sitting"):ti,ab OR ("short bouts of accumulated exercise"):ti,ab OR (SBAE):ti,ab OR (breaks):ti,ab OR ("exercise snacks"):ti,ab |
| #2 | Search: ("Sedentary Behavior"):ti,ab,kw OR ("Sitting Position"):ti,ab,kw OR ("sedentary"):ti,ab,kw OR ("Sedentary Behavior"):ti,ab,kw OR ("sedentary behaviors"):ti,ab,kw OR ("sedentary behaviour"):ti,ab,kw OR ("sedentary behaviours"):ti,ab,kw OR ("sedentary time"):ti,ab,kw OR ("prolonged sedentary time"):ti,ab,kw OR ("sedentariness"):ti,ab,kw OR ("sitting"):ti,ab,kw OR ("sitting time"):ti,ab,kw OR ("time spent sitting"):ti,ab,kw OR ("time sitting"):ti,ab,kw OR ("seated time"):ti,ab,kw OR ("prolonged sitting"):ti,ab,kw OR ("uninterrupted sitting"):ti,ab,kw OR ("continuous sitting"):ti,ab,kw OR ("seated rest"):ti,ab,kw |
| #3 | Search: ("Cognition"):ti,ab OR ("Executive Function"):ti,ab OR ("Attention"):ti,ab OR ("Memory"):ti,ab OR ("Learning"):ti,ab OR ("Intelligence"):ti,ab OR ("Reaction Time"):ti,ab OR ("Psychomotor Performance"):ti,ab OR (cognit*):ti,ab OR ("cognitive performance"):ti,ab OR ("cognitive function"):ti,ab OR ("cognitive functions"):ti,ab OR ("cognitive functioning"):ti,ab OR (neurocognit*):ti,ab OR ("neuro-cognitive"):ti,ab OR ("Executive Function"):ti,ab OR ("executive functions"):ti,ab OR ("executive functioning"):ti,ab OR ("executive control"):ti,ab OR ("cognitive control"):ti,ab OR ("Attention"):ti,ab OR (attentional):ti,ab OR (attentiveness):ti,ab OR ("attentional control"):ti,ab OR ("attention control"):ti,ab OR ("working memory"):ti,ab OR (updating):ti,ab OR ("updating memory"):ti,ab OR ("task switching"):ti,ab OR ("switch task"):ti,ab OR ("set shifting"):ti,ab OR ("cognitive flexibility"):ti,ab OR ("processing speed"):ti,ab OR ("information processing"):ti,ab OR ("Reaction Time"):ti,ab OR ("reaction times"):ti,ab OR ("response time"):ti,ab OR ("response times"):ti,ab OR ("psychomotor vigilance"):ti,ab OR (PVT):ti,ab OR (Stroop):ti,ab OR (Flanker):ti,ab OR ("go/no-go"):ti,ab OR ("go/no-go"):ti,ab OR ("n-back"):ti,ab OR (nback):ti,ab OR ("digit span"):ti,ab OR ("trail making"):ti,ab OR ("digit symbol substitution"):ti,ab OR (DSST):ti,ab OR ("symbol digit"):ti,ab OR (SDMT):ti,ab OR ("choice reaction time"):ti,ab OR ("simple reaction time"):ti,ab |
| #4 **Combine** | #1 AND #2 AND #3 |

**Supplementary Materials 3. The details of cognitive assessment tools of included studies**

**Supplementary Table 3**. The measurement tools for different outcomes

| **Cognitive domains** | **Measurement tools of interests** |
| --- | --- |
| **Executive Function** | （1）Stroop Color Word Test（***SCWT***）；（2）Trail Making Test（***TMT***）；（3）Modified Flanker Task（***MFT***）；（4）Modified Stroop Colour-Word Task（***MSCWT***）；（5）Verbal Fluency Tasks（***VFT***）；（6）Flanker Task（***FT***）；（7）Set-Shifting Test（***SST***）；（8）Task Switching（***TS***）；（9）Serial-7 Subtractions（***S7S***）；（10）Serial Three Subtraction Task（***STS***）；（11）Groton Maze Learning Test（***GMLT***）；（12）Comprehensive Trail Making Test（***CTMT***）；（13）Eriksen Flanker Task（***EFT***）；（14）Trail Making Test Part B（***TMT-B***）；（15）Test of Attentional Performance 2.3（***TAP 2.3***） |
| **Memory Function** | （1）Two-Back Task（***2-back***）；（2）One Card Learning Task（***OCLT***）；（3）One-Back Task（***1-back***）；（4）n-back（***N-back***）；（5）Letter Memory Task（***LMT***）；（6）Figural Intersections Task（***FIT***）；（7）Test of Attentional Performance 2.3（***TAP 2.3***） |
| **Information Processing** | （1）Identification Task（***IDN***）；（2）Rapid Visual Information Processing（***RVIP***）；（3）Discrete Simple Reaction Time（***DSRT***）；（4）Numeric Vigilance（***NV***）；（5）Detection Test（***DT***） |
| **Attention** | （1）Choice Reaction Time（***CRT***）；（2）Simple Reaction Time（***SRT***）；（3）D2 Attention Test（***D2T***）；（4）Test of Attentional Performance 2.3（***TAP 2.3***）；（5）Detection Test（***DT***）；（6）Computerised Mental Performance Assessment System（***COMPASS***）；（7）Test of Attentional Performance（***TAP***） |

**Supplementary Materials 4.** **Summary Figure of Overall and Subgroup Effects of sitting interruptions**

**Supplement Figure 1.** Overall and subgroup effects of sitting interruptions on Information Processing. NOTE: In the left panel, each green horizontal line represents the 95% CI for an individual effect size, and the yellow point indicates the corresponding Hedges’ g estimate. The red diamond indicates the overall pooled effect, and the black horizontal line indicates the 95% prediction interval (PI). In the right-hand subgroup panels, small points represent individual effect sizes, whereas larger points with black horizontal lines represent subgroup estimates and their 95% CIs. Values to the left of zero favour prolonged sitting, whereas values to the right of zero favour sitting interruptions.

**Supplement Figure 2.** Overall and subgroup effects of sitting interruptions on Attention. NOTE: In the left panel, each green horizontal line represents the 95% CI for an individual effect size, and the yellow point indicates the corresponding Hedges’ g estimate. The red diamond indicates the overall pooled effect, and the black horizontal line indicates the 95% prediction interval (PI). In the right-hand subgroup panels, small points represent individual effect sizes, whereas larger points with black horizontal lines represent subgroup estimates and their 95% CIs. Values to the left of zero favour prolonged sitting, whereas values to the right of zero favour sitting interruptions.

**Supplementary Materials 5. The forest plots of meta-analysis**

**Supplement Figure 3.** Forest Plot of Global Cognition

**Supplement Figure 4.** Forest Plot of Information Processing

**Supplement Figure 5.** Forest Plot of Executive Function

**Supplement Figure 6.** Forest Plot of Memory Function

**Supplement Figure 7.** Forest Plot of Attention.

**Supplementary Materials 6. Subgroup Analysis Results**

**Supplementary Table 4**. Subgroup Analysis of the Effects of sitting interruptions on Executive Function

| **Subgroup** | ***K* (*N)*** | ***Hedges’g*** | ***95%CI*** | ***P_d_*** | ***P_m_*** |
| --- | --- | --- | --- | --- | --- |
| **Age** |  |  |  |  | **0.041** |
| Young adults | 83（11） | 0.136 | 0.059 to 0.214 | < 0.001 |  |
| Middle-aged adults | 7（3） | 0.064 | -0.161 to 0.288 | 0.572 |  |
| Older adults | 5（2） | 0.415 | 0.199 to 0.630 | < 0.001 |  |
| **BMI** |  |  |  |  | **0.049** |
| Normal weight | 77（11） | 0.134 | 0.055 to 0.213 | 0.001 |  |
| Overweight/Obesity | 18（5） | 0.265 | 0.101 to 0.434 | 0.002 |  |
| **Type** |  |  |  |  | **< 0.001** |
| Walking | 61（11） | 0.122 | 0.039 to 0.205 | 0.004 |  |
| Standing | 8（1） | 0.483 | 0.138 to 0.828 | 0.006 |  |
| Stair climbing | 3（1） | -0.269 | -0.625 to 0.086 | 0.136 |  |
| Resistance | 17（3） | 0.113 | -0.053 to 0.279 | 0.181 |  |
| Jiggling | 5（1） | 1.121 | 0.739 to 1.501 | < 0.001 |  |
| Cycling | 1（1） | 0.465 | -0.116 to 1.046 | 0.116 |  |
| **Setting** |  |  |  |  | **0.050** |
| Simulated | 8（1） | 0.484 | 0.135 to 0.834 | 0.007 |  |
| Laboratory | 87（15） | 0.145 | 0.071 to 0.218 | < 0.001 |  |
| **Intensity** |  |  |  |  | 0.070 |
| Moderate | 42（6） | 0.087 | -0.018 to 0.192 | 0.104 |  |
| Low | 53（10） | 0.218 | 0.122 to 0.315 | < 0.001 |  |

Note: ***K*** indicates the number of effect sizes/comparisons, and ***N*** indicates the number of studies included in each subgroup. ***BMI*** = body mass index; ***CI*** = confidence interval; ***P_d_*** = p value for the pooled effect within each subgroup; ***P_m_*** = p value for differences between subgroups.

**Supplementary Table 5**. Subgroup Analysis of the Effects of sitting interruptions on Memory Function

| **Subgroup** | ***K* (*N*)** | ***Hedges’g*** | ***95%CI*** | ***P_d_*** | ***P_m_*** |
| --- | --- | --- | --- | --- | --- |
| **Age** |  |  |  |  | **<0.001** |
| Adolescents | 13（2） | 0.041 | -0.206 to 0.289 | 0.738 |  |
| Young adults | 21（4） | 0.318 | 0.108 to 0.529 | 0.004 |  |
| Middle-aged adults | 16（3） | 0.039 | -0.194 to 0.271 | 0.739 |  |
| Older adults | 3 （1） | 1.468 | 1.070 to 1.866 | <0.001 |  |
| **BMI** |  |  |  |  | **0.004** |
| Normal weight | 27（5） | 0.037 | -0.182 to 0.256 | 0.738 |  |
| Overweight/Obesity | 26（5） | 0.499 | 0.278 to 0.721 | <0.001 |  |
| **Type** |  |  |  |  | **0.041** |
| Walking | 22（5） | 0.345 | 0.105 to 0.585 | 0.006 |  |
| Standing | 10（1） | 0.687 | 0.264 to 1.110 | 0.002 |  |
| Stair climbing | 6 （1） | -0.066 | -0.535 to 0.402 | 0.776 |  |
| Resistance | 13（2） | 0.322 | -0.286 to 0.370 | 0.797 |  |
| Cycling | 2（1） | 0.150 | -0.618 to 0.918 | 0.696 |  |
| **Setting** |  |  |  |  | 0.954 |
| Simulated | 22（3） | 0.257 | -0.012 to 0.527 | 0.061 |  |
| Laboratory | 31（7） | 0.267 | 0.054 to 0.480 | 0.015 |  |
| **Intensity** |  |  |  |  | **0.004** |
| Moderate | 20（4） | 0.065 | -0.138 to 0.268 | 0.523 |  |
| Low | 33（6） | 0.541 | 0.303 to 0.780 | <0.001 |  |

Note: ***K*** indicates the number of effect sizes/comparisons, and ***N*** indicates the number of studies included in each subgroup. ***BMI*** = body mass index; ***CI*** = confidence interval; ***P_d_*** = p value for the pooled effect within each subgroup; ***P_m_*** = p value for differences between subgroups.

**Supplementary Table 6**. Subgroup Analysis of the Effects of sitting interruptions on Information Processing

| **Subgroup** | ***K* (*N*)** | ***Hedges’g*** | ***95%CI*** | ***P_d_*** | ***P_m_*** |
| --- | --- | --- | --- | --- | --- |
| **Age** |  |  |  |  | 0.769 |
| Young adults | 17（3） | 0.365 | 0.008 to 0.723 | 0.046 |  |
| Older adults | 1（1） | 0.195 | -0.962 to 1.352 | 0.726 |  |
| **BMI** |  |  |  |  | 0.207 |
| Normal weight | 8（2） | 0.128 | -0.355 to 0.609 | 0.583 |  |
| Overweight/Obesity | 10（2） | 0.530 | 0.097 to 0.962 | 0.019 |  |
| **Type** |  |  |  |  | 0.441 |
| Walking | 10（3） | 0.195 | -0.243 to 0.634 | 0.355 |  |
| Standing | 3（2） | 0.463 | -0.378 to 1.305 | 0.257 |  |
| Resistance | 2（1） | 0.215 | -0.747 to 1.172 | 0.639 |  |
| Cycling | 3（1） | 0.933 | 0.075 to 1.790 | 0.035 |  |
| **Setting** |  |  |  |  | **0.037** |
| Simulated | 9（1） | 0.597 | 0.139 to 1.055 | 0.014 |  |
| Laboratory | 9（3） | 0.142 | -0.277 to 0.562 | 0.482 |  |
| **Intensity** |  |  |  |  | 0.211 |
| Moderate | 6（1） | 0.093 | -0.477 to 0.665 | 0.732 |  |
| Low | 12（3） | 0.475 | 0.077 to 0.872 | 0.022 |  |

Note: ***K*** indicates the number of effect sizes/comparisons, and ***N*** indicates the number of studies included in each subgroup. ***BMI*** = body mass index; ***CI*** = confidence interval; ***P_d_*** = p value for the pooled effect within each subgroup; ***P_m_*** = p value for differences between subgroups.

**Supplementary Table 7**. Subgroup Analysis of the Effects of sitting interruptions on Attention

| **Subgroup** | ***K* (*N*)** | ***Hedges’g*** | ***95%CI*** | ***P_d_*** | ***P_m_*** |
| --- | --- | --- | --- | --- | --- |
| **Age** |  |  |  |  | 0.056 |
| Young adults | 11（3） | 0.109 | -0.152 to 0.369 | 0.378 |  |
| Middle-aged adults | 2（1） | 0.013 | -0.437 to 0.462 | 0.952 |  |
| Older adults | 1（1） | -0.49 | -0.884 to -0.097 | 0.019 |  |
| **BMI** |  |  |  |  | 0.070 |
| Normal weight | 11（3） | 0.109 | -0.154 to 0.371 | 0.386 |  |
| Overweight/Obesity | 3（2） | -0.261 | -0.570 to 0.047 | 0.086 |  |
| **Type** |  |  |  |  | 0.951 |
| Walking | 9（3） | 0.016 | -0.291 to 0.323 | 0.912 |  |
| Resistance | 3（1） | -0.079 | -0.675 to 0.518 | 0.776 |  |
| Cycling | 2（1） | 0.012 | -0.556 to 0.582 | 0.962 |  |
| **Intensity** |  |  |  |  | 0.212 |
| Moderate | 8（2） | 0.114 | -0.199 to 0.427 | 0.104 |  |
| Low | 6（3） | -0.151 | -0.454 to 0.153 | 0.301 |  |

Note: ***K*** indicates the number of effect sizes/comparisons, and ***N*** indicates the number of studies included in each subgroup. ***BMI*** = body mass index; ***CI*** = confidence interval; ***P_d_*** = p value for the pooled effect within each subgroup; ***P_m_*** = p value for differences between subgroups.

**Supplementary Materials 7. Meta-Regression Analysis Results**

**Supplement Figure 8.** Meta-regression analyses of moderators of Information Processing. NOTE: Meta-regression plots show associations between continuous moderators and information processing effect sizes. Panels represent age, BMI, inter-bout interval, female participants, bout duration, and total session duration. Each point represents one effect size. The solid purple line indicates the fitted meta-regression line, and the shaded band represents the 95% confidence interval. The horizontal dashed line marks the null effect (g = 0). Marginal histograms show the distributions of moderator values along the top axis and effect sizes along the right axis, expressed as the number of effect sizes (No. of ES). Each panel reports the regression coefficient (β), P value, and number of effect sizes (k).

 **Supplement Figure 9.** Meta-regression analyses of moderators of Attention. NOTE: Meta-regression plots show associations between continuous moderators and attention on effect sizes. Panels represent age, BMI, inter-bout interval, female participants, bout duration, and total session duration. Each point represents one effect size. The solid purple line indicates the fitted meta-regression line, and the shaded band represents the 95% confidence interval. The horizontal dashed line marks the null effect (g = 0). Marginal histograms show the distributions of moderator values along the top axis and effect sizes along the right axis, expressed as the number of effect sizes (No. of ES). Each panel reports the regression coefficient (β), P value, and number of effect sizes (k).

**Supplementary Materials 8. Sensitivity Analysis Results**

**8.1 Sensitivity to the assumed correlation coefficients**

**Supporting Comment R2-6.** Two correlation coefficients are assumed at different stages of the analysis and serve distinct statistical purposes. (1) A paired-difference correlation (primary r = 0.5) is used to reconstruct the sampling variance of each within-participant effect size in the crossover trials. (2) A within-study dependency correlation (primary r = 0.6) is used to impute the off-diagonal (covariance) elements of the sampling variance–covariance matrix that links multiple effect sizes from the same study. Tables SX and SY vary these two coefficients in turn. Across both, the pooled estimates, their 95% confidence intervals, and the resulting conclusions are essentially unchanged, indicating that the findings are robust to the assumed correlations.

**Supplement Figure 10.** Sensitivity of pooled effects to the assumed correlation coefficients across cognitive domains.

**8.2 Domain-specific leave-one-out sensitivity**

**Supplement Figure 11.** Global Cognition

**Supplement Figure 12.** Information Processing

**Supplement Figure 13.** Executive Function

**Supplement Figure 14.** Memory Function

**Supplement Figure 15.** Attention

**8.3 Domain-specific within-study correlation sensitivity (r = 0.4/0.6/0.8)**

**Supplement Figure 16.** Global Cognition

**Supplement Figure 17.** Executive Function

**Supplement Figure 18.** Memory Function

**Supplement Figure 19.** Information Processing

**Supplement Figure 20.** Attention

**Supplementary Materials 9. Publication Bias and Statistical Power**

**Supplement Figure 21.** Funnel plots and power analyses.

**Supplementary Materials 10. GRADE Evidence Certainty Assessment**

**Supplementary Table 8.** GRADE evidence profile for acute cognitive effects of brief standing or physical activity breaks during prolonged sitting

| Outcome | Participants (RCTs) | **GRADE Evidence Certainty Assessment** | | | | | Hedge’s *g* (95% CI) | Certainty* |
| --- | --- | --- | --- | --- | --- | --- | --- | --- |
|  |  | Risk of bias | Inconsistency | Indirectness | Imprecision | Other concerns |  |  |
|  | | | | | | | | |
| **Global Cognition** | **28**  **(2 RCT)** | **Some serious** | **Not serious** | **Not serious** | **Serious** | **Risk of publication bias** | **0.57 (-0.20 to 1.34)** | **⨁◯◯◯ Very low** |
| **Executive**  **Function** | **355**  **(16 RCT)** | **Some serious** | **Not serious** | **Not serious** | **Not Serious** | **Risk of publication bias** | **0.18 (0.03 to 0.32)** | **⨁⨁◯◯ Low** |
| **Memory**  **Function** | **218**  **(10 RCT)** | **Some serious** | **Not serious** | **Not serious** | **Serious** | **None** | **0.37(0.13 to 0.61)** | **⨁⨁◯◯ Low** |
| **Information Processing** | **99**  **(4 RCT)** | **Some serious** | **Not serious** | **Not serious** | **Serious** | **None** | **0.32（-0.21 to 0.86）** | **⨁⨁◯◯ Low** |
| **Attention** | **135**  **(5 RCT)** | **Some serious** | **Not serious** | **Not serious** | **Serious** | **None** | **-0.08（-0.38 to 0.22）** | **⨁⨁◯◯ Low** |
| † **GRADE levels of certainty**  High: We are very confident that the true effect lies close to the estimate.  Moderate: The true effect is likely close to the estimate, but there is a possibility it is substantially different.  Low: Our confidence in the effect estimate is limited—the true effect may be substantially different.  Very low: We have very little confidence in the effect estimate. | | | | | | | | |
